# LTR retroelements are intrinsic components of transcriptional networks in frogs

**DOI:** 10.1186/1471-2164-15-626

**Published:** 2014-07-23

**Authors:** José Horacio Grau, Albert J Poustka, Martin Meixner, Jörg Plötner

**Affiliations:** Dahlem Center for Genome Research and Medical Systems Biology, Fabeckstraße 60-62, 14195 Berlin, Germany; Museum für Naturkunde Berlin, Leibniz-Institut für Evolutions- und Biodiversitätsforschung, Invalidenstraße 43, 10115 Berlin, Germany; Max-Planck-Institut für Molekulare Genetik, AG Evolution and Development, Ihnestraße 73-75, 14195 Berlin, Germany; Services in Molecular Biology GmbH, Rudolf-Breitscheid-Str. 70, 15562 Rüdersdorf, Germany

**Keywords:** LTR retroelements, *Silurana*, *Pelophylax*, Anura, RNAseq, Transcriptome, Embryogenesis

## Abstract

**Background:**

LTR retroelements (LTR REs) constitute a major group of transposable elements widely distributed in eukaryotic genomes. Through their own mechanism of retrotranscription LTR REs enrich the genomic landscape by providing genetic variability, thus contributing to genome structure and organization. Nonetheless, transcriptomic activity of LTR REs still remains an obscure domain within cell, developmental, and organism biology.

**Results:**

Here we present a first comparative analysis of LTR REs for anuran amphibians based on a full depth coverage transcriptome of the European pool frog, *Pelophylax lessonae*, the genome of the African clawed frog, *Silurana tropicalis* (release v7.1), and additional transcriptomes of *S. tropicalis* and *Cyclorana alboguttata.* We identified over 1000 copies of LTR REs from all four families (Bel/Pao, Ty1/Copia, Ty3/Gypsy, Retroviridae) in the genome of *S. tropicalis* and discovered transcripts of several of these elements in all RNA-seq datasets analyzed. Elements of the Ty3/Gypsy family were most active, especially Amn-san elements, which accounted for approximately 0.27% of the genome in *Silurana*. Some elements exhibited tissue specific expression patterns, for example Hydra1.1 and MuERV-like elements in *Pelophylax*. In *S. tropicalis* considerable transcription of LTR REs was observed during embryogenesis as soon as the embryonic genome became activated, i.e. at midblastula transition. In the course of embryonic development the spectrum of transcribed LTR REs changed; during gastrulation and neurulation MuERV-like and SnRV like retroviruses were abundantly transcribed while during organogenesis transcripts of the XEN1 retroviruses became much more active.

**Conclusions:**

The differential expression of LTR REs during embryogenesis in concert with their tissue-specificity and the protein domains they encode are evidence for the functional roles these elements play as integrative parts of complex regulatory networks. Our results support the meanwhile widely accepted concept that retroelements are not simple “junk DNA” or “harmful genomic parasites” but essential components of the transcriptomic machinery in vertebrates.

**Electronic supplementary material:**

The online version of this article (doi:10.1186/1471-2164-15-626) contains supplementary material, which is available to authorized users.

## Background

Transposable elements (TEs) are mobile genetic elements that constitute large portions of the genome in eukaryotes [1, 2]. In primates including humans, for example, about 50% of the genome consists of TEs [3]. Vast genome size differences among species are directly related to the TE content [1, 2, 4, 5]; thus TE abundance and diversity are characteristic features of plant and animal genomes [6].

Transposable elements play an important role for genome organization and evolution as substantial providers of large scale mutation events, creating genetic variability that natural selection can act upon [1]. They can affect both single genes and entire genomes [7, 8] by chromosomal rearrangements including insertions, duplications, deletions, and recombination events [9, 10]. Although most TE-caused mutations are expected to be deleterious, some are neutral or even adaptive. TE-derived sequences such as promoters [11–15], polyadenylation signals and termination sites [16–18], and smRNAs [19] are involved in regulation of gene expression at both the transcriptional and post-transcriptional level [2, 9, 20]. In addition, TE proliferation is thought to create new regulatory networks and to participate in the rewiring of pre-established regulatory networks [2].

Little is known about the regulation of TE activity. Large scale elimination and suppression of retroelements have both been documented for the genome of the pufferfish [21]. Several factors have been shown to be responsible for TE silencing, such as RNAi [22, 23], especially by piRNAs [24, 25], and DNA methylation [26].

In some cases activation of TEs seems to be environmentally mediated. There is evidence, for example, that retrotransposition activates the expression of stress response genes thus providing a positive feedback under stressful conditions to promote survival related genes [27].

Transposable elements are generally classified into Class I elements (called retrotransposons or retroelements), which use an RNA intermediate for transposition; and Class II elements, which replicate without an RNA intermediate, either by a cut-and-paste mechanism (DNA transposons), by rolling circle DNA replication (helitrons), or by so far unknown mechanisms (politrons/mavericks). Among the Class I elements two major subclasses are recognized: (1) retroelements (REs) with long terminal repeats (LTRs) and (2) elements without LTRs (non-LTR REs) [20, 28]. In this study we focus on LTR REs, which can be classified into four major families, namely Bel/Pao, Ty1/Copia, Ty3/Gypsy, and retroviruses [29, 30]. A common LTR retrotransposon typically encodes two polyproteins, termed GAG and POL. The group-specific antigen (GAG) usually contains matrix, capsid, and nucleocapsid domains; POL consists of aspartic proteinase (AP), reverse transcriptase (RT), ribonuclease (RN), and integrase (INT) domains, the latter three (RT, RN, INT) are responsible for retrotranscribing cDNA from RNA intermediates and inserting it into the host genome.

Endogenous retroviruses (ERVs) constitute a specific class of LTR REs that additionally contain an open reading frame (ORF) for an envelope protein (ENV), which enables ERVs to move from one cell to another. In contrast, all other LTR REs either lack or contain a remnant of an ENV gene and can only reinsert into their own host genome [1, 31, 32]. There are, however, ERVs that secondary lost their ENV gene and thus their infectious ability. Such ERVs are retrotransposing instead of infecting other cells as do typical retroviruses [33].

As a precondition for understanding the role of LTR REs in shaping genomes the diversity of these elements has to be systematized [34–36] . For this purpose several computer programs have been developed to automatically detect LTR REs [37]. Some of these computing methods have made it possible to detect and identify previously unknown elements [38]; however, only a few comprehensive studies on LTR RE diversity have been carried out on non-model organisms. Furthermore, many genomes still host remnants of inactive retrotransposons corresponding to ancient retrotransposition events. These “genomic fossils” have accumulated mutations through time; many of them are difficult to identify because they have lost some of their characteristic features, thus making them imperceptible to automatic searches.

In this study we analyze the abundance and diversity of LTR retrotransposons found in the genome of the western clawed frog *Silurana* (*Xenopus*) *tropicalis* and compare it to a full depth coverage transcriptome of an advanced frog species, the European pool frog *Pelophylax* (*Rana*) *lessonae*. Amphibians are a very important evolutionary link between lunged and gilled vertebrates; they are also amongst the animals with the largest genomes [39]. The sequencing of the *Silurana* genome revealed a high diversity of TEs, even higher than in many other eukaryotes and vertebrates studied, including all four major families of LTR REs [40], thus making the frog genomic and transcriptional landscapes excellent environments to study the variability and dynamics of LTR REs. We were able to effectively estimate the abundance of the LTR RE families and clades within the *Silurana* genome, systematized them into clades on the basis of phylogenetic analyses, which we then used to analyze the diversity and expression patterns of LTR REs in the transcriptional landscapes of different tissues obtained from *P. lessonae*, *S. tropicalis*, and of eight individuals of *Cyclorana alboguttata*.

Based on RNAseq data we show that certain elements are tissue-specific expressed and for the first time that the expression patterns of ERVs change during embryonic development of *Silurana*. Finally, we discuss factors that may affect the transcription of LTR REs in the context of tissue- and genome-specificity.

## Results

### Transcriptome assembly

Four transcriptomes were assembled. The largest transcriptome comprised the libraries of *Silurana* developmental stages [41], which spanned 148 million bp and 247 thousand sequences with an N50 of 791. The largest assembled sequence originated from the *P. lessonae* transcriptome and consisted of 94519 bp, it included an ORF of 93336 bp coding for 31122 amino acids (aa), a full length frog ortholog of titin (Gr. titan = giant), the largest known vertebrate gene/protein. The presence of this unusually long transcript indicates the good assembly quality of the *P. lessonae* transcriptome.

### LTR RE diversity and abundance in the *Silurana*genome

**Figure 1:**
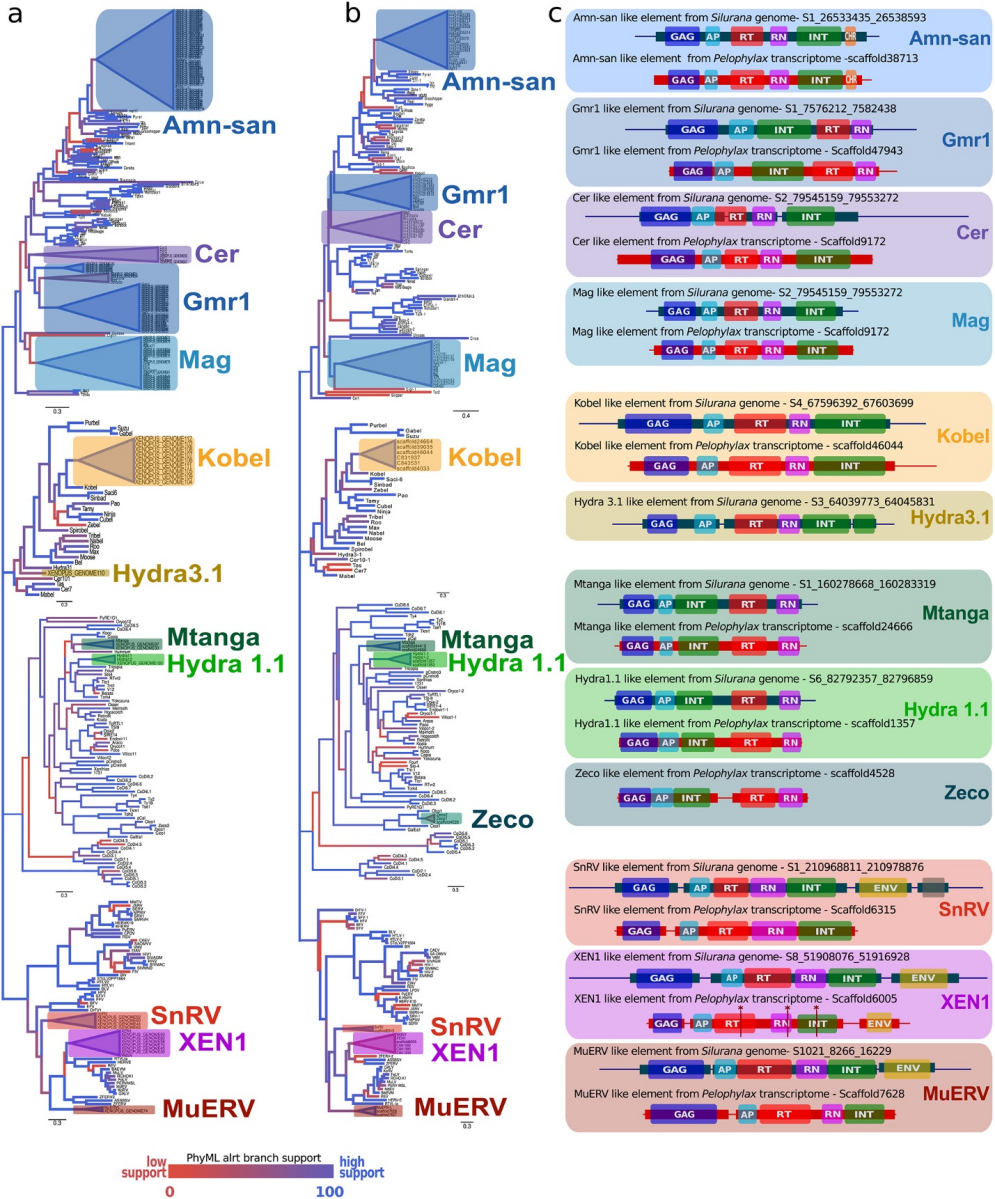
**Classification and structure of LTR retroelements in the frog genome and transcriptomes.** Maximum-likelihood (ML) trees calculated on the basis of 256 known RT domains of eukaryotic LTR REs including amino acid sequences obtained from the *Silurana tropicalis* genome **(a)** and the transcriptomes of *Pelophylax lessonae* **(b)**. Diagrammatic presentation of LTR REs **(c)** found in the *Silurana* genome (blue) and in the transcriptome of *P. lessonae* (red). The thin lines represent the overall length of the retroelement including the LTRs, while thick bars depict open reading frames for aspartic proteinase (AP), chromo domain (CHR), envelope protein (ENV), group-specific antigen (GAG), integrase (INT), RNase (RN), and reverse transcriptase (RT). Frameshifts are indicated by asterisks (*).

Phylogenetic reconstructions (Figure 1, Additional file 1: Figures S1-S5) based on RT domains, revealed the presence of LTR REs of all four classes (Bel/Pao, Ty1/Copia, Ty3/Gypsy, Retroviridae) in the genome and transcriptomes of *S. tropicalis* and the transcriptomes of *P. lessonae* and *C. alboguttata* (Table 1). We were able to identify at least eleven types of LTR REs (Figure 1, Table 1), some of them either unknown or else previously neglected in the *Silurana* genome.

**Table 1:** **LTR retroelements detected in the genome of** ***Silurana tropicalis***

Family	Type	GSM1 (genomic ORFs)	GSM2 (LTR-harvest)	AAE	AEL [bp]	[%]
Bel/Pao	Kobel	129	140	135	7000	0.06468
	Hydra3.1	0	2	1	7000	0.00048
Ty1/Copia	Hydra1.1	6	8	7	4000	0.00192
	Mtanga	8	8	8	4000	0.00220
Ty3/Gypsy	Amn-san	749	805	777	5000	0.26688
	Cer	30	25	28	7000	0.01322
	Gmr	177	215	196	8000	0.10772
	Mag	65	102	84	4000	0.02294
Retroviridae	MuERV	2	1	2	6000	0.00062
	SnRV	7	11	9	10000	0.00618
	XEN1	7	12	10	10000	0.00653
**Total**		**1180**	**1329**	**1257**		**0.49337**

Based on the results of two genome search methods (GSM1 and 2) the average amount of elements (AAE), the average element length (AEL), and the percentage [%] of the elements in the genome were calculated.

Two types of **Bel/Pao** elements (Kobel and Hydra3.1) were found in the *Silurana* genome (Table 1). A Kobel-like element was present in multiple copies (135) in the *Silurana* genome; it was transcriptionally active in *Silurana, Pelophylax*, and *Cyclorana* (Figure 1, Table 2). Hydra 3.1-like elements were present with 2 copies in the *Silurana* genome but absent in the frog transcriptomes analyzed.

**Table 2:** **LTR retroelements discovered in the genome of** ***Silurana tropicalis*** **(SIL-G) and different transcriptomes of** ***S. tropicalis*** **(SIL-T: adult tissues; SIL-D: developmental stages),** ***Cyclorana alboguttata*** **(CYC-T: adult individuals), and** ***Pelophylax lessonae*** **(PEL-T: adult tissues) with remarks on the occurrence and distribution of these elements among animals, plants, and fungi**

Family	Type	Occurrence/remarks	Ref.	Genome	Transcriptome			
				SIL-G	SIL-T	SIL-D	CYC-T	PEL-T
**Bel/Pao**	Kobel	first detected in the genome of the hemichordate *Saccoglossus kowalevskii*; present in protostomes and deuterostomes	[32]	●	●	●	●	●
Only known from animal genomes; relatively few elements are reported across diverse animal phyla								
	Hydra3.1	described from the genome of *Hydra magnipapillata*; also present in cnidarian and protostome genomes	[42]	●				
**Ty1/Copia**	Hydra1.1	includes two elements that have described from the invertebrate *Hydra magnipapillata* and the zebrafish *Danio rerio*	[32]	●	●	●		●
Widespread in eukaryotic genomes; two main sub-clades can be distinguished								
	Mtanga	so far only known from the genome of the mosquito *Anopheles gambiae*	[43]	●				●
	Zeco	restricted to crustaceans, urochordates, and fish	[44]					●
**Ty3/Gypsy**	Amn-san	belongs to the vertebrate lineage of chromoviruses, active in fish, amphibians, and reptiles	[32]	●	●	●	●	●
The largest family of LTR REs; widespread among the genomes of plants, animals, and fungi								
	Cer	first described from nematodes	[45]	●				●
	CsRN1	characterized from the genome of the trematode *Clonorchis sinensis*	[46]				●	
	Gmr	circulate within the genomes of deuterostomes; characterized by Ty1/Copia pol-domain organization	[47, 48]	●	●	●	●	●
	Mag	widely spread through animal genomes including vertebrates	[49, 50]	●	●	●	●	●
**Retroviridae**	MuERV	poorly known outside mammals (belongs to class 3 of retroviruses)	[51, 52]	●		●		●
Exclusively found in vertebrate genomes; characterized by the presence of a gene encoding an envelope protein								
	SnRV	described from the snakehead fish (*Ophicephalus striatus*); belongs to class 1 of retroviruses	[53]	●	●	●		●
	XEN1	described from *Xenopus laevis*; belongs to class 1 of retroviruses	[54]	●	●	●	●	●

Three types of **Ty1/Copia** elements (Hydra1.1, Mtanga, Zeco) were found in the frog genome and transcriptomes (Figure 1, Tables 1 and 2). Hydra1.1 and Mtanga-like elements were detected in the *Silurana* genome with 6 and 8 copies, respectively. Zeco-like elements, however, were found only in the transcriptome of *P. lessonae* together with transcripts of Hydra1.1- and Mtanga-like elements.

We found four types of **Ty3/Gypsy** elements (Amn-san, Cer, Gmr1, Mag) in the *Silurana* genome (Table 1). In total we identified over 700 copies of Amn-san elements, about 30 copies of Cer-like elements, ca. 200 copies of Gmr1-like elements, and approximately 80 copies of Mag-like elements. Multiple transcripts of these elements were also found in *Pelophylax*, *Silurana*, and *Cyclorana* tissues (Table 2).

Among the **Retroviridae** elements, three types (Murine Endogenous Retrovirus-like element, MuERV; Snakehead fish retrovirus, SnRV; and *Xenopus laevis* endogenous retrovirus, XEN1) were found in the *Silurana* genome and the frog transcriptomes analyzed (Figure 1, Tables 1 and 2; Additional file 1: Tables S1 and S2). A MuERV-L was present in 1-2 copies in the *Silurana* genome and in the *P. lessonae* transcriptome. Moreover, we were able to locate about 9 copies of SnRV-like elements within the *Silurana* genome and recovered a complete ENV-less element of this virus in the *P. lessonae* transcriptome. A XEN1 was present in the *Silurana* genome with ca. 10 copies and several transcripts were present in the transcriptomes of *Pelophylax, Silurana,* and *Cyclorana* (Table 2).

### Genome colonization and proliferation of LTR elements

**Figure 2:**
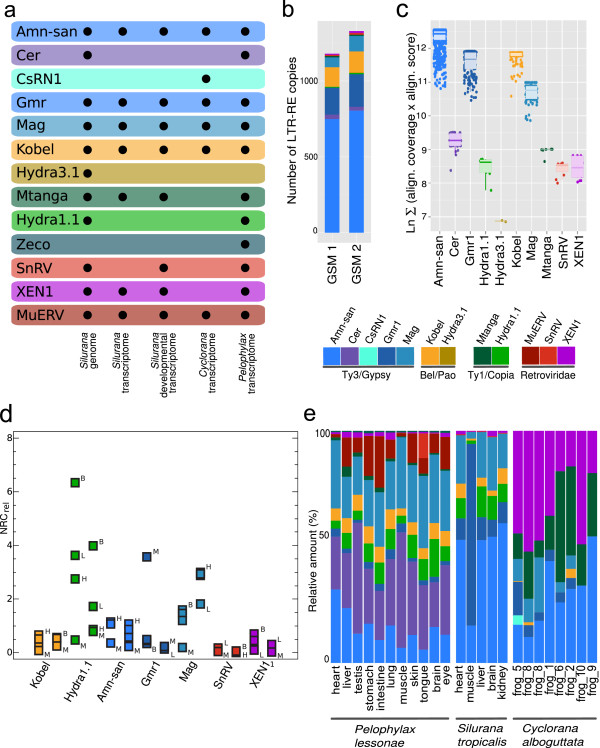
**Diversity and expression patterns of LTR retroelements in the frog genome and transcriptomes. (a)** Diversity of LTR REs in the genome of *Silurana* and in the frogs transcriptomes analyzed. **(b)** Number of LTR RE copies in the *Silurana* genome; **(c)** Proliferation patterns based on average relatedness of LTR REs in the *Silurana* genome. The average relatedness was calculated on the basis of amino acids as LOG (∑ (Alignment coverage *Alignment score)), in which a higher relatedness score indicates that the elements within that group are closer related to one another. **(d)** Arithmetic means of relative NRC values calculated for brain (B), heart (H), liver (L), and muscle (M) of *S. tropicalis* (left points) and *P. lessonae* (right points). **(e)** Relative amount of LTR REs in different frog transcriptomes.

The diversity of LTR REs is largely the same in *Silurana* and *Pelophylax* (Figure 2a). There is evidence, however, that at least two elements (Zeco and Hydra3.1) have been acquired or lost since their last common ancestor. Our results clearly demonstrate that Ty3/Gypsy and Bel/Pao are the most prolific LTR RE families within the *Silurana* genome (Figure 2b), while elements of Ty1/Copia and Retroviridae show less success in fixation. Among all frog LTR REs, Amn-san elements are the most abundant, with multiple genomic copies (>700) followed by Gmr1 and Kobel (Table 2); some of the copies show very low sequence divergence as indicated by the average relatedness values calculated on the basis of the nucleotide and aa sequences of the RT domain (Figure 2c).

### Transcript abundance and differential expression

Our results clearly show that LTR REs from all four families (Bel/Pao, Ty1/Copia, Ty3/Gypsy, Retroviridae) are differentially transcribed. Ty3/Gypsy appears to be the most active LTR RE family as indicated by both the number of copies and NRC (Normalized Read Count) values (Table 2, Additional file 1: Tables S1 and S2).

In adult individuals of *Silurana* and *Pelophylax*, the expression of some elements exhibit tissue specific patterns (Figure 2d); significant differences in expression were observed for three elements (Amn-san, Gmr1, Mag) in *Silurana* and for two elements (Hydra1.1, MuERV) in *Pelophylax* (Additional file 1: Figures S6 and S7; Tables S1, S2, and S4). Hydra1.1, for example, exhibited the highest relative NRC values in brain and lowest in muscle transcriptomes in both *Pelophylax* and *Silurana* (Figure 2d, Additional file 1: Figure S7). It is also noticeable that SnRV is over-expressed in the tongue tissue of *P. lessonae* showing a circa 5 time higher relative NRC value than in the other tissues investigated (Additional file 1: Figure S6). In muscle of both *Silurana* and *Pelophylax* most elements were on average less expressed than in other tissues (Additional file 1: Figure S7). Muscle tissues of eight *C. alboguttata* individuals, however, showed only little similarity in both the relative amount and diversity of transcribed LTR REs (Figure 2e).

**Figure 3:**
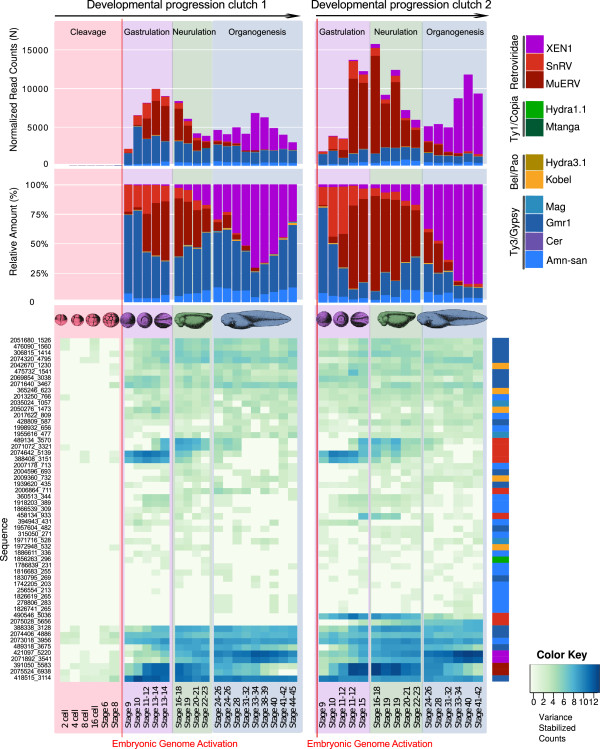
**Normalized read counts and relative amount of expression of LTR retroelement (LTR RE) transcripts throughout the developmental progression of** ***S. tropicalis*** **progession.** The presence of each type of LTR RE found within the transcriptome of *S. tropicalis* throughout 23 distinct developmental stages is summarized.

In the embryonic development of *S. tropicalis* transcription of LTR REs begins as soon as the embryonic genome is activated, ca. 6-8 hours after insemination of eggs at developmental stage 8.5, i. e. at the midblastula transition (MBT) [55], Figure 3; here stage 8.5 is included in stage 9). While Ty3/Gypsy, Bel/Pao, and Ty1/Copia elements did not show clear differential expression patterns during embryonic development, retroviral elements, particularly MuERV and SnRV, were most actively transcribed during gastrulation and neurulation, and XEN1 during organogenesis.

### LTR RE annotations

Predicted LTR REs from the *Silurana* genome and LTR RE transcripts from all frog transcriptomes exhibited many ORFs which contained protein domains normally associated with retrotranscription of LTR REs and their reinsertion into the genome. The preliminary annotation of these genomic elements further revealed specific domains for each type of LTR RE that are linked with cell regulation in animals (Table 3).

**Table 3:** **Examples of protein domains found in LTR REs predicted from the genome of** ***Silurana tropicalis*** **which might play a role in gene regulation and transcriptional networking**

Domain	Pfam No.	Included in	Domain description/function	Ref.
CHROMO (Chromatin organization modifier)	pfam00385	This domain was exclusively found within Amn-san elements. Circa 80% of these elements within the *Silurana* genome encode for a chromo domain.	The chromo domain is about 40–50 amino acids long. It is contained in various proteins involved in chromatin remodeling and the regulation of gene expression in eukaryotes during development.	[56–58]
PNMA (Para-neoplastic antigen MA)	pfam14893	Found so far in about 30% of Cer elements and in about 16% of Gmr elements.	This protein domain has so far only been studied in mammals, where it has been associated with neurological disorders.	[59, 60]
			Because of the homology between PNMA proteins and an apoptosis inducing protein (MOAP1), the involvement of PNMA proteins in apoptosis is hypothesized.	
SCAN	pfam02023 pfam00096	This domain was found in over 50% of Gmr elements only.	The SCAN domain family of Zinc finger transcription factors, they are thought to be implicated in regulating genes involved in lipid metabolism, cell survival, and differentiation.	[61]
Exo/endo phosphatase	pfam03372 pfam14529	Found in about 12% of Mtanga elements.	The exo-/endonuclease phosphatase family of proteins includes magnesium dependent endonucleases and a large number of phosphatases involved in intracellular signaling.	[62, 63]
Zinc Fingers Zf-H2C2_2 (Zinc-finger double domain) Zf-CCHC (Zinc knuckle)	pfam13465	About 40% of SnRVs contained a Zinc-finger double domain; about 20% of XEN1 elements contained a Zinc knuckle.	Zinc finger (Znf) domains are relatively small but very diverse protein motifs which can target specific molecules. Znf-containing proteins function in gene transcription, translation, mRNA trafficking, cytoskeleton organization, epithelial development, cell adhesion, protein folding, chromatin remodeling, and Zinc sensing, to name but a few.	[64, 65]
	pfam00098		A Zinc knuckle is a Zinc binding motif of the general structure CX2CX4HX4C where X can be any amino acid. The motifs mostly originate from retroviral gag proteins (nucleocapsid). Zinc knuckles are involved in eukaryotic gene regulation.	
UBN2 gag-polypeptide of LTR copia-type	pfam14223	Found in Copia-type elements, in about 30% of Mtanga elements and in about 80% of Hydra1.1 elements.	Ubinucleins are members of a protein family that contain a conserved HIRA binding domain which interacts with the N-terminal WD repeats of HIRA/Hir proteins. UBN1 and UBN2 are believed to be the orthologs of Hpc2p, a subunit of a nucleosome assembly complex in budding yeast (HIR), involved in regulation of histone gene transcription.	[66]

Pfam: Protein family database.

## Discussion

### LTR retroelement diversity in the genomic and transcriptomic landscapes of frogs

Based on two different genomic search methods we have found between 1200 and 1300 LTR REs of four distinct families within the *Silurana* genome, containing at least LTRs and a retrotranscriptase ORF. LTR elements, however, constitute only a small fraction of total nuclear *Silurana* DNA compared to non-LTR REs and DNA transposons, which comprise up to one third of the *Silurana* genome [40]. Calculating the average length of each element and multiplying the average number of each element (Table 2), it can be suggested that around 0.49% (ca. 7.18 Mbp) of the *Silurana* genome assembly 7.1 (in total 1.45 billion bp) is composed of LTR REs. This estimation is concordant to the 7.43 Mbp calculated by Smit et al. [67] using the Repeat Masker *Silurana* genomic dataset (available at http://www.repeatmasker.org/genomes/xenTro2/RepeatMasker-rm327-db20090202/xenTro2.fa.out.gz) but differs from the value (9%) published by Hellsten et al. [40]; this discrepancy probably reflects a lower threshold used by Hellsten et al. to identify LTR REs.

Besides elements typical for vertebrate genomes such as Amn-san, Gmr1, and retroviruses, we have identified LTR REs of the Ty1/Copia and Bel/Pao clades, which have so far only been found in the genomes of phylogenetically distant aquatic animals. The Hydra3.1 element, for example, was first described from the genome of a freshwater animal *Hydra magnipapillata;* Kobel-like elements are known from the genomes of basal protostomes and deuterostomes [32].

Amn-san elements were most abundant in all of our data sets. They account for about 0.27% of the genome size in *Silurana* and can be considered as the most successful LTR REs in the *Silurana* genome. This assumption is evidenced by the coexistence of multiple copies with high sequence similarity, speaking for relatively recent bursts in activity of one or even several active master elements or recurrent genomic invasions. Besides closely related Amn-san elements, we found copies with higher sequence divergences that may trace back to older and now inactive elements. Large numbers of LTR REs have also been found in the giant genomes of salamanders, primarily Ty3/Gypsy elements [68], which supports our results that these LTR REs, particularly Amn-san, are the most numerous elements and account for nearly half of the LTR RE content in the *Silurana* genome. Moreover, our *Silurana* genomic dataset contained twice as much Bel/Pao elements as had been previously reported by de la Chaux and Wagner [35], who used a more selective pipeline and different reference sequences to identify LTR REs.

### Colonization of the amphibian genome by LTR REs

Very little is known about genome colonization by LTR REs or about their evolutionary dynamics which is thought to encompass both gradual and vertical processes, as well as distinct modular, salutatory, and reticular events [32]. As indicated by the similar LTR RE spectrum in the genomes of *Silurana* and *Pelophylax*, most of the REs were already present in the genome of their last common ancestor, which presumably lived ca. 230 million years before present [69]. It can be assumed, however, that genome colonization by LTR REs predates the split between Rhinophrynidae + Pipidae and Neobatrachians because members of all RE families except Retroviridae are widely distributed among the genomes of plants, fungi, and animals [32].

LTR REs are usually inherited vertically from generation to generation; there is also evidence for a horizontal transfer of such elements between species [70–74]. A successful spread of LTR REs assumes a stable integration into the germline of the host, which can be achieved when eggs or early embryonic stages are infected. The underlying transfer requires a vector; it was speculated that parasites may transmit nuclear DNA including TEs [74, 75]. The mechanisms of the transmission process, however, remain obscure. In this context it should be noted that Cer elements found in the genome of *Silurana* and the transcriptome of *Pelophylax* showed closest relationships to elements described from the genome of the nematode *Caenorhabditis elegans* [45] *.* We do not know whether these Cer elements originated directly from frog genomes or from the genomes of putative parasites. The latter possibility is more parsimonious because highest expression of Cer elements was observed in muscle and testis; both tissues are known to be colonized by parasitic flatworms [76, 77].

### Differential expression of LTR REs

The expression of LTR REs in vertebrates is thought to depend on a variety of genetic and epigenetic factors as indicated by specific spatiotemporal expression patterns, i.e. differences in the expression profiles of distinct elements (families) between tissues, sexes, ontogenetic and age stages, individuals, and species [78–82]. Tissue-specific expression patterns of single LTR REs, especially Hydra1.1 and MuERV, have been observed in the frog transcriptomes analyzed. The most enigmatic example for tissue-specific expression is the Snakehead retrovirus (SnRV), which was highly expressed in the tongue of *P. lessonae* but at very low levels in the other tissues investigated. The significance of this pattern is not yet understood just as this ERV is not well studied either.

Similar patterns of cell type specific expression have been reported for the ZFERV virus of the zebrafish; for this ERV the thymus appears to be a major tissue for retroviral activity [78]. Pervasive, tissue-specific RE transcription is likely to have functional consequences on the protein-coding transcriptome [80] and is thought to be directly linked to the role these elements may play in physiology of organs [78, 79].

Evidence for individual differences of LTR RE expression comes from the *Cyclorana* dataset; here a small number of Kobel-like elements were transcribed in muscle tissue of only some individuals. This suggests that expression of LTR REs may play a role in the process of individual adaptation and may affect phenotypic variability. Because the *Silurana* transcriptomic datasets are pooled from several specimens [41], individual effects should be minimized as indicated by similar expression profiles of LTR RE transcripts in *S. tropicalis* eggs and embryos obtained from two different clutches (Figure 3). Moreover, there is evidence for species-specific expression of LTR REs. For example, XEN1-like elements exhibited only minor transcription in *Pelophylax* and *Silurana*, but were relatively highly expressed in the muscle tissue of *Cyclorana* compared to the other elements*.*

Our analyses clearly demonstrate that LTR REs are differentially expressed during ontogenetic development of *S. tropicalis*; there are clear transitions between three LTR RE communities at particular stages of development. Transcription starts abruptly at the MBT (stage 8.5, Figure 3). Before the MBT *Silurana* embryos undergo 12 rapid synchronous cleavages; this phase is also characterized by the absence of cell motility. At the MBT the blastomers become motile and the cell cycle becomes more complex. While low levels of transcription are known to occur before the MBT, especially of genes associated with phosphorylation, the cell cycle, signal transduction, and apoptosis [41, 83–85] we did not find significant expression of viral-related transcripts before stage 8.5. The significant change of LTR RE transcription profiles during embryogenesis indicates that LTR REs are probably involved in cell differentiation and organogenesis in *S. tropicalis* as has already been demonstrated by Sinzelle et al. [81] for the ERV XTERV1.

For mammals there is increasing evidence that LTR REs are involved in gene regulation and developmental processes. In mouse oocytes and preimplantation embryos, for example, retroviruses exhibited a high contribution to the maternal mRNA pool and different LTR REs had specific, developmentally regulated expression patterns [86]. In a 2-cell (2C) stage embryo cDNA library prepared by Peatson et al. [87], the bulk of interspersed repeat ESTs were MuERV, similar to the situation observed in gastrulation and neurulation stages of *Silurana*. In mice the 2C stage is the critical phase when the embryo switches from a maternal to a zygotic transcriptome [88] comparable to the MBT in *Silurana* [89]. In mouse 2C-like embryonic stem cells (ESCs) the expression pattern of murine ERV elements with leucine t-RNA primer (MuERVL) overlapped with more than 100 2C-specific genes that have co-opted regulatory elements from these retroviruses to initiate their transcription [90]. More than 25% of the nearly 700 MuERVL copies were activated, and 307 genes generated chimeric transcripts with junctions to MuERVL elements. Similar observations were obtained from human ESCs in which HERV-H was highly expressed but became silenced on differentiation into embryoid bodies [91]. Based on these results it can be suggested that ERVs may have an important gene regulatory role already in early mammalian development by contributing to the specification of cell types.

In contrast to the mouse genome, only 1-2 MuERV copies were found in the genome of *Silurana* where they were highest expressed from stage 13-14 (mid gastrulation) to stage 22-23 (end of neurulation). One of these copies carried an ORF of unknown function and an ENV protein.

During embryonic development LTR REs operate as alternative promotors, enhancers [13–15, 92], first exons for a subset of host genes [87], and as targets of transcription factors [93]. Retroelements are even able to serve host functions for genes over longer distances as the example of the human ERV-9 demonstrates [94]. The LTR/POL II complex of this ERV appears to mediate the long range transfer of proteins from the LTR to the ß-globolin gene. Moreover, RE derived mRNAs are important sources for small RNAs, which are known to be necessary for regulation of gene expression [95].

Based on the fact that LTR REs are apparently involved in key and early stages of embryonic development in *Silurana,* we hypothesize that LTR REs including ERVs, were already exapted as regulators of embryonic development in lower vertebrates, i.e. long before the earliest mammalian genomes evolved.

### LTR REs as evolvability toolboxes

There is increasing evidence that LTR REs have greatly contributed to generate the adaptive genetic diversity observed in living organisms [96, 97]. Beside the fact that LTR REs are common components of transcriptional networks, the protein domains they carry are known to be essential for genome maintenance and dynamics such as transcription regulation, mRNA trafficking, intracellular signaling, cell survival, and differentiation [15]. LTR REs typically include highly specific RNA binding domains (Zinc fingers, Zinc knuckles, SCAN domains) [61, 64, 65]; domains for catalysis of DNA integration into the genome (integrase domain); peptide cleavage (pepsin-like aspartases and protease domains), RNA and DNA cleavage (RNAse domain, endonuclease domain) [62, 63]; and reverse transcription (retrotranscriptase domain); in addition some carry group specific antigens (GAG domains) [98]; chromatin organization modifiers (chromo domains) [56, 57]; and trans-membrane glycoproteins (ENV domain). The domain composition is element-specific (Table 3), for example chromo domains were only found within Am-san elements; more than half of the Gmr elements exclusively contained a SCAN domain, while Zinc finger and Zinc knuckle domains were only identified in retroviruses. Moreover, LTR RE derived glycoproteins, in particular from ERVs, are thought to act as blocking receptors against exogenous infective viruses (a phenomenon called retroviral interference or super-infection resistance) [99, 100].

A not yet discussed putative function concerns the ENV domain of ERVs which is responsible for cell entry [101] and also has an immunosuppressive function [102]. We found that the ENV domain of MuERV was only expressed during embryogenesis but not in adult tissues of *Silurana*. This fact indicates that MuERV still possesses the capability to overcome cell membranes during embryogenesis and predisposes one to believe that ERVs might play a general role in signal transduction pathways and thus for coordination and regulation of ontogenetic processes in frogs and probably also in other vertebrates. Because of the relative low copy number of ERVs in the *Silurana tropicalis* genome (<25), this species could serve as a suitable model to study the effects ERVs have on ontogenesis and cell differentiation.

Taking the known and putative functions of ERVs and remnant LTR elements into consideration, the common view that they have to be considered as fossil representatives of retroviruses extant at the time of their insertion into the germline [15, 103] has to be questioned. Because complex phenomena such as molecular orchestration of embryonic development, placentation, and immunity are closely accompanied by ERVs and their derivatives we are more inclined to believe that LTR REs and in general TEs significantly contributed to the rise and diversification of vertebrate animals.

## Conclusions

We here present the first comprehensive study on the diversity of LTR REs in frog genomes. We found LTR REs of all four families (Bel/Pao, Ty1/Copia, Ty3/Gypsy, Retroviridae) in the genome of *Silurana* and in the transcriptional landscapes of *Silurana* and *Pelophylax*. Ty3/Gypsy and Bel/Pao are the most abundant LTR RE classes within the frog genome and transcriptome. Amn-san elements from the Ty3/Gypsy class are the most prolific with over 700 full-length genomic copies. It has been shown that LTR REs are differentially transcribed not only across different tissues of the same frog, but also across different species of frogs and across different individuals of the same species. Differential expression of LTR REs occurred also during the embryonic development of *Silurana,* where transcription of LTR REs begins as soon as the embryonic genome is activated, followed by clear transitions between three LTR RE communities at particular stages of development. Their involvement in key and early stages of development suggests that LTR REs, especially ERVs, were already exapted as regulators of embryonic development in lower vertebrates, i.e. before the earliest mammalian genomes evolved.

Measured in terms of the huge amount and variability of LTR REs, only little is known on their specific genomic functions. Therefore, experimental approaches are urgently needed to better understand the roles LTR REs play for cell function, gene regulation, and organismic development, separately and in concert with other genes and genetic factors. Future efforts should also include studies focused on the functions of the protein domains encoded within each LTR RE type, and particularly the ENV domain of ERVs.

Beside the fact that LTR REs are transcriptionally active, their cell type-specificity and differential expression during ontogenetic development emphasize once again their importance for organismic development in vertebrates as intrinsic components of regulatory networks.

## Methods

### Tissue preparation, RNA isolation, and *de novo*sequencing

Organs (brain, heart, eye, intestine, liver, lung, muscle, skin, stomach, testes, tongue) for tissue samples were taken from two *Pelophylax lessonae* males (PL68-2012, PL74-2012) collected near Melzow, Germany (53°11'00"N, 13°54'00"E), snap frozen in liquid nitrogen and stored at -80°C. RNA and DNA was isolated simultaneously from each tissue mentioned above using the AllPrep DNA/RNA Mini Kit (Qiagen, Cat.No. 80204). Frozen tissue pieces were disrupted using mortar and pestle, and homogenized in RLT buffer in TissueLyser for 2 min at 20 Hz. RNA quantification and integrity were determined using a Qubit® 2.0 Fluorometer (Life Technologies, Cat.No. Q32866) and a 2100 BioAnalyser (Agilent Technologies, Cat.No. G2940CA), respectively, according to the manufacturer’s instructions.

MRNA-seq libraries were prepared from 2000 ng of total RNA using TruSeq RNA Sample Prep Kit v2 (Illumina, Cat.No. RS-122-2001) with a modification of the protocol allowing to preserve directional information about the transcripts [104]. First, mRNA was isolated within a pool of total RNA and chemically fragmented. Then double-stranded (ds) cDNA synthesis was performed with the incorporation of dUTP in the second strand. The ds cDNA fragments were further processed following a standard Illumina sequencing library preparation scheme: end polishing, A-tailing, adapter ligation, and size selection. Prior to final library amplification, the dUTP-marked strand was selectively degraded by Uracil-DNA-Glycosylase (UDG). The remaining strand was amplified to generate a cDNA library suitable for sequencing. Paired-end 2x50 bp sequencing was carried out on the Illumina HiSeq2000 platform, generating on average 50 million paired-end reads or 2.5 GB per sample.

### Genome data sources and *de novo*assembly of transcriptome data

The genome assembly (release v7.1) of *Silurana tropicalis* was downloaded from Xenbase.org [105] [date last accessed 29 July 2014]: ftp://ftp.xenbase.org/pub/Genomics/Tropicalis_Scaffolds/7.1/xenopus_tropicalis_v7.1.tar.gz.

To study the transcriptional diversity and dynamics of LTR REs in frogs we assembled transcriptomes of *P. lessonae* and *S. tropicalis* from several tissues. *P. lessonae* transcriptomes of brain, eye, intestine, liver, lung, skin, stomach, testis, and tongue originated from individual PL74-2012, transcriptomes of heart and muscle from individual PL68-2012. Transcriptomes for brain, liver, kidney, heart, and skeletal muscle of *S. tropicalis* are based on publicly available RNA-seq datasets (Accession No. SRX191164-68, 5 runs, 39 Gbases). Additionally, we assembled a transcriptome by using a dataset of 23 distinct developmental stages of *S. tropicalis* from two egg clutches ( [41]; Accession No. SRA051954 - 40 runs compromising 92 Gbases) to study the dynamics of LTR REs through embryonic development. Finally, eight RNA-seq libraries from muscle tissue samples of eight individuals of the Australian green-striped burrowing frog *Cyclorana alboguttata* (Accession No. SRA059487 - 8 runs, 42 Gbases) were analyzed to answer the question whether the expression of LTR elements is individual-specific.

All SRA files were converted to fastq format using the fastq-dump utility of the SRA tool kit (available from NCBI) and transcriptome data were assembled with SOAPdenovo-trans [106]. We assembled the transcriptomes of *Cyclorana* and of the developmental stages of *Silurana* using different k-mer lengths (k = 23, 31, 51), merged the contig files and constructed a non-redundant file using the program CD-HIT [107, 108].

### *Pelophylax*deep transcriptome assembly

Prior to *de novo* sequence assembly, an inhouse python script was used to clean raw Illumina reads from adapter sequences (on average 1-3%) and low quality reads (Phred score below 11). Reads containing Ns were excluded. On average about 10% of the sequences were excluded by this procedure. A total of 1,119,579,890 reads was assembled simultaneously using SOAPdenovo-trans; settings (other than default) used were –K 31 –M3 –F –G 200 (per default up to five transcripts per locus were allowed).

### LTR retroelement identification

**Figure 4:**
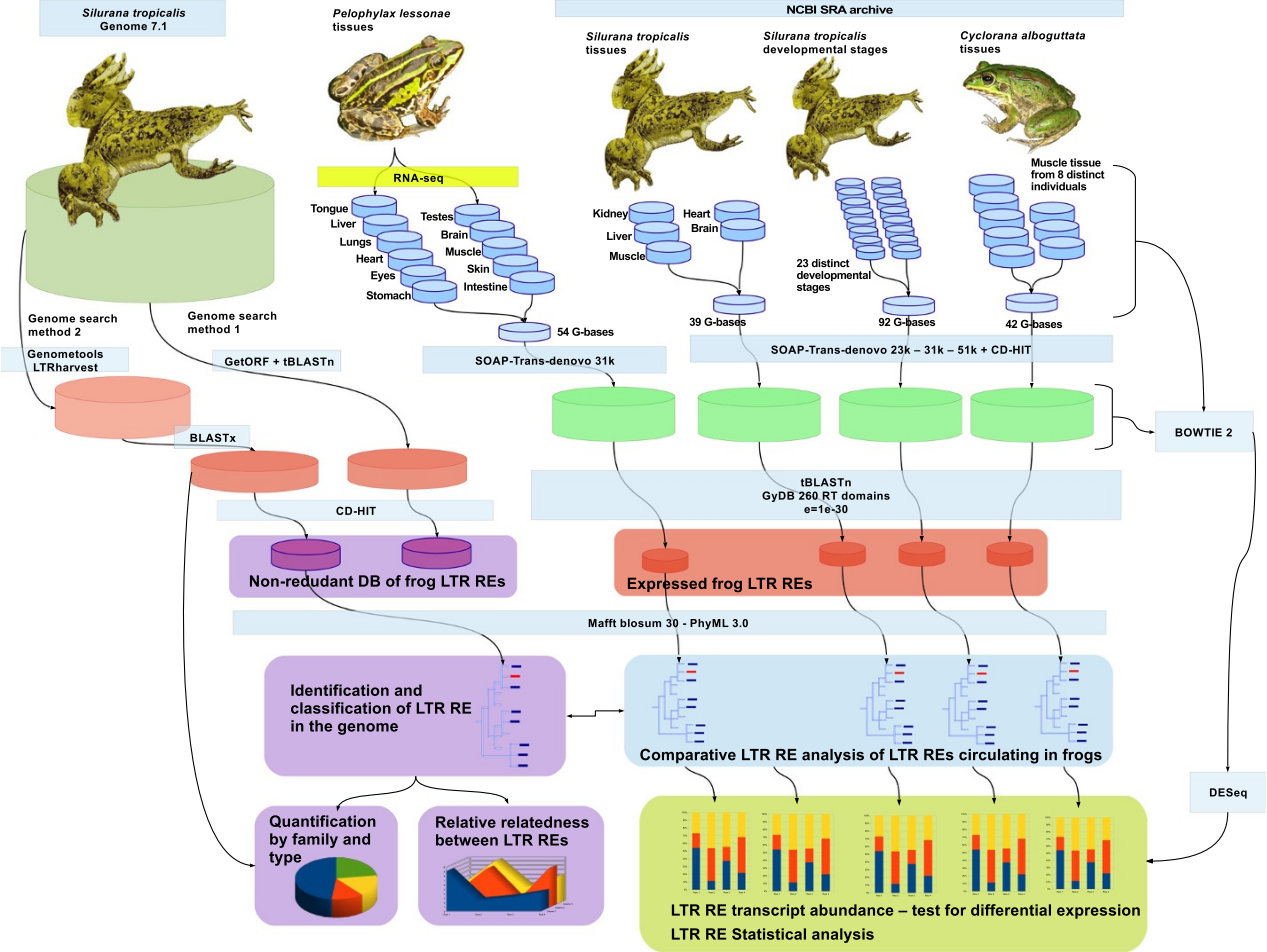
**Work flow diagram summarizing data flow from sequencing to statistical analysis.** Abbreviations used in the Figure: DB, database; LTR REs, long terminal repeat retroelements.

We created several datasets to gain independent overviews on LTR REs in each frog transcriptome and in the *Silurana* genome (Figure 4). In all searches we relied heavily on a reference collection of retroelement domains and alignments obtained from the publicly available Gypsy Database 2.0 (GyDB) [36]. For the detection of LTR REs we used the retro-transcriptase (RT) domain because it is the best conserved through evolutionary time [109]. In order to obtain a custom representation of the LTR RE diversity, including all four LTR RE families occurring within the frog genome and transcriptomes, the following methods were applied:

*Genome LTR RE search method 1*: We used tblastn to query the complete RT domains of GyDB against the entire *Silurana* genome reporting matches with an e-value of 1e-40 and alignments for the 10,000 best matches.

*Genome LTR RE search method 2:* We applied the program suffixerator, which is part of GenomeTools (http://genometools.org) with default parameters and created an enhanced suffix file which was later scanned with LTR harvest [110], a *de novo* detector of LTR REs, with relaxed parameters (-seed 20, minlenLTR 30, maxlenLTR 2000, similar 70) to predict more LTR REs. To leave out false LTR RE predictions, we then searched each LTR harvest predicted sequence against a database of RT domains of GyDB using blastx. Matches with an e-value of 1e-40 and alignments for only the best match were reported.

*Transcriptome LTR RE search method:* For the identification of LTR REs in the transcriptomes we used blastx to query each transcriptome sequence against the RT domains of GyDB. All sequences with e-values of 1e-30 were considered to belong to LTR REs.

### Systematic classification

Because the results from both genome search methods yielded thousands of RT alignments, we separately clustered each genome LTR RE dataset using the program CD-HIT with an identity threshold of 80%, and discarded sequences shorter than 120 aa to reduce the high number of similar and identical copies of each retroelement.

Databases resulting from single frog transcriptomes and from the S*. tropicalis* genome were analyzed separately. We fused each dataset with the complete RT domains of GyDB, aligned the sequences, and inferred a Maximum-Likelihood (ML) tree in order to accurately place the retroelements in a phylogenetic context. All alignments were conducted with the program Mafft [111] using local alignment and a Blosum 30 aa substitution matrix as parameters. Final alignment files were prepared by removing columns with more than 70% of gaps (Additional file 2). ML trees were calculated with the program PhyML 3.0 [112] using 4 rate categories and a nearest neighbor interchange (NNI) tree search. Branch support was estimated with an approximate likelihood ratio test (aLRT) as implemented in PhyML.

The ML trees based on the different genome search methods (1 and 2) were largely the same. We selected the tree resulting from the LTR harvest predictions (method 2). To check the integrity, i.e. the completeness, of the LTR REs, we used NCBI’s Conserved Domain Database [113] and a custom query databases derived from the GyDB. Candidate sequences and regions were extracted and queried against a references database containing the GAG, POL, dUTPASE, and CHR domains of each class of LTR REs.

### LTR retroelement quantification

In order to estimate the quantity of LTR RE copies for each type that coexist within the *Silurana* genome we applied two different counting procedures: (1) all ORFs with a minimal length of 450 bp were translated into aa between the START and STOP codons using EMBOSS getorf [114]; the resulting protein predictions were blasted against a custom database containing only RT domains of LTR REs previously distinguished in the phylogenetic analysis. (2) The second method based on the results of LTR harvest (genome search method 2). We also searched against a selected database of RT domains and counted the amount of hits accumulated for each element.

### Proliferation analysis

In order to determine which of the elements have been more efficient in copying and inserting themselves within the *Silurana* genome, we used the inner regions (regions without LTRs) that resulted from LTR harvest (genome search method 2), separated each LTR RE prediction by element type based on the previous analyses and queried each group of elements against itself by using Blast. We blasted the aa region (Blastp) of the RT domain as well as the whole inner regions (Blastn) of LTR RE predictions using default parameters.

After processing the Blast reports we were able to estimate the relatedness of each element within its group by extracting the alignment score and coverage. For each element we normalized the relatedness value using the formula: element relative relatedness = Ln ∑ (alignment coverage × alignment score).

### LTR annotation

To predict putative functions of LTR REs we annotated the genomic copies as well as the transcripts from all frog transcriptomes analyzed. We translated all ORFs using EMBOSS getorf [114] with default parameters and the option '-find 1' which translates only regions between the start and stop codon. The resulted protein predictions were then classified by their domains using Hmmer [115] and Pfam-A reference databases [116]. Domain hits with e-values of 1e-10 were parsed out (Additional files 3 and 4).

### Transcript abundance and tests for differential expression

As a first step we treated the assembled transcriptomes as a reference genome and mapped the read library of each tissue against the transcriptome using Bowtie 2.1.0 [117] with default options and settings to report the 20 best alignments of every read with the -K 20 parameter. Raw count data were obtained through a custom python script and analyzed with DEseq [118] to normalize count data across tissues (Additional file 1: Figures S8-S11). Based on these normalized read counts (NRC) expression patterns of different LTR REs were analyzed for each transcriptome (Additional file 1: Tables S1 and S2). For tissue-specific transcriptomes we also calculated relative values of normalized read counts (NRC_rel_) dividing the single NRC values by their arithmetic mean (Additional file 1: Table S3). Based on these NRC_rel_ values we compared tissue-specific expression of all LTR REs detected (Additional file 1: Figures S6 and S7, Table S4). Because NRC and NRC_rel_ values were not normally distributed, a LOG transformation or POWER transformation based on the method of Box and Cox [119] was applied (Additional file 1). Transformed data were tested for normality and variance homogeneity using the test statistics of Shapiro-Wilk [120] and Levene [121], respectively. NRC and NRC_rel_ values were analyzed with the One-Way ANOVA procedure and/or the Kruskal-Wallis test [122] to determine significant differences in the expression patterns (Additional file 1: Tables S1, S2, and S4). Statistical calculations were done with the program Statgraphics Centurion Version 15.2.14 (Statpoint Technologies, Inc., Warrenton, Virginia, USA).

### Data access

RNA-seq libraries for the eleven tissues of the *Pelophylax lessonae* deep transcriptome study are available from SRA sequence database under accession number SRP036849.

### Ethics

Our animal use protocols follow the Animal Welfare Act of the Federal Republic of Germany and the recommendations contained in "Guidelines for Use of Live Amphibians and Reptiles in Field Research" compiled by the American Society of Ichthyologists and Herpetologists (ASIH), the Herpetologists League (HL), and the Society for the Study of Amphibians and Reptiles (SSAR). All experiments in this study were performed under the ethical permits RS7/SPN176 and LUGV_RO7-4610/73#81213/2012 which were approved by Landesumweltamt Brandenburg, Regionalabteilung Süd, Referat RS7 Naturschutz and Landesamt für Umwelt, Gesundheit und Verbraucherschutz, Brandenburg, Regionalabteilung Ost, respectively.

## Electronic supplementary material

Additional file 1: **Supplemental material consists of supplementary methods, figures, and tables.** (PDF 3 MB)

Additional file 2: **Aligned amino acid datasets and phylogenetic trees.** Amino acid sequence alignments and tree files for the different frog transcriptomes and the *Silurana* genome. (ZIP 193 KB)

Additional file 3: **Annotation of LTR RE predicted from the genome of** ***Silurana tropicalis.*** Table listing Pfam-A hits of ORFs contained in LTR RE predictions of the *Silurana tropicalis* genome*.* (CSV 365 KB)

Additional file 4: **Annotation of LTR RE transcripts identified in the frog transcriptomes used in this study.** Table listing Pfam-A hits of ORFs contained in LTR RE transcripts of several frog transcriptomes*.* (CSV 22 KB)
