# Supplementary material for: LTR retroelements are intrinsic components of transcriptional networks in frogs
Source: BMC Genomics. 2014 Jul 23;15:626. doi: 10.1186/1471-2164-15-626 (PMC4131045; doi:10.1186/1471-2164-15-626)

Grau, J., A. Poustka, M. Meixner, and J. Plötner

# LTR retrotransposons are intrinsic components of transcriptional networks in frogs
## Supplemental Material

**1. Phylogenetic relationships of LTR retroelements detected in *Silurana tropicalis, Pelophylax lessonae* and *Cyclorana alboguttata*. (Figures S1-S5).**

**Figure S1.** Maximum-likelihood tree inferred on the basis of 256 known retrotranscriptase domains from eukaryotes and LTR-retroelements from the *Silurana tropicalis* genome (obtained by genome search method 2). Branch support was calculated using approximate likelihood ratio tests as implemented in PhyML 3.0. Branch labels in green correspond to LTR-retroelements identified in the frog genome.

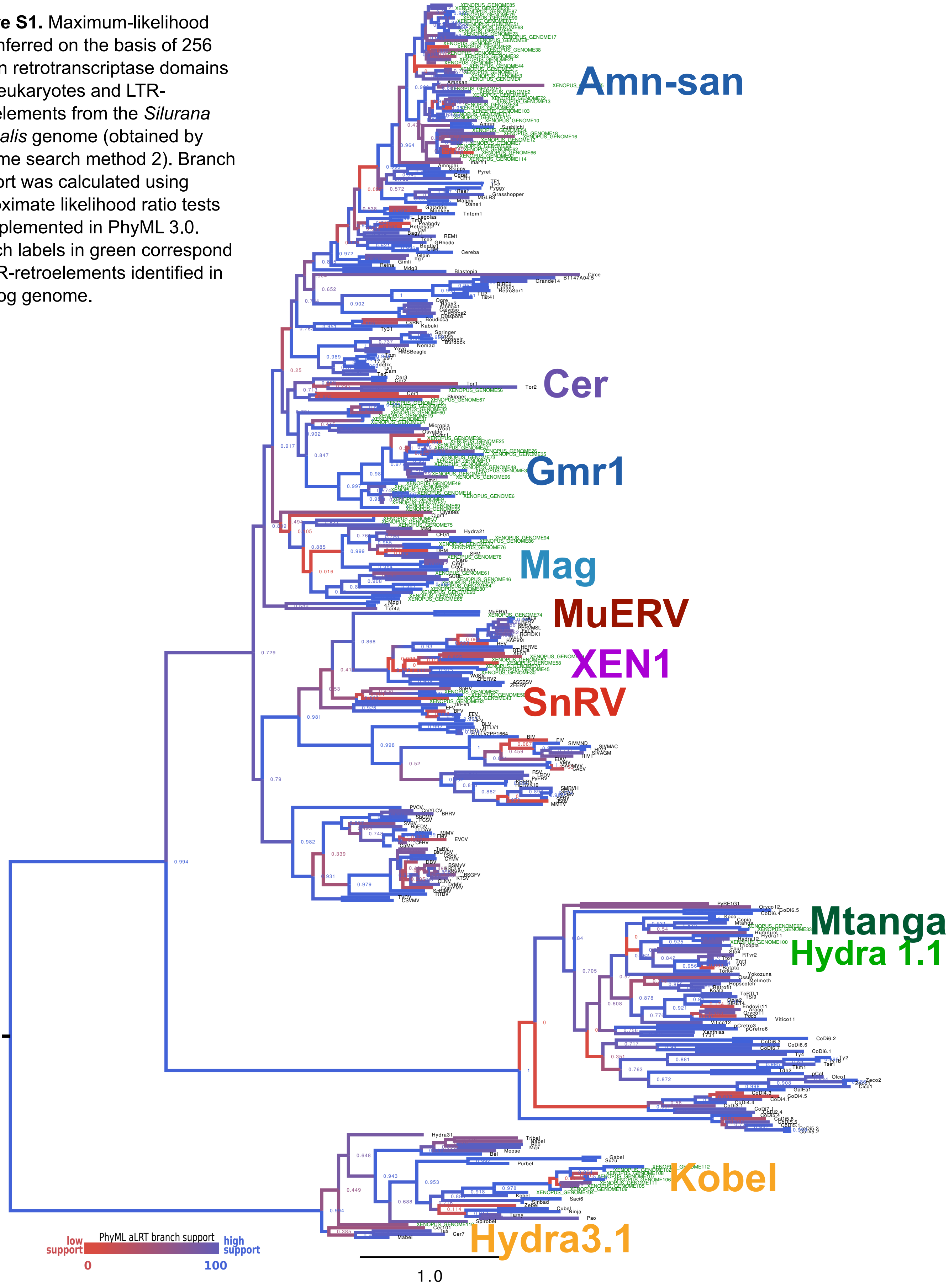

**Figure S2.** Maximum-likelihood tree inferred on the basis of 256 known retrotranscriptase domains from eukaryotes and LTR-retroelements from the *Pelophylax lessonae* transcriptome. Branch support was calculated using approximate likelihood ratio tests as implemented in PhyML 3.0. Branch labels in green correspond to LTR-retroelements identified in the frog transcriptome.

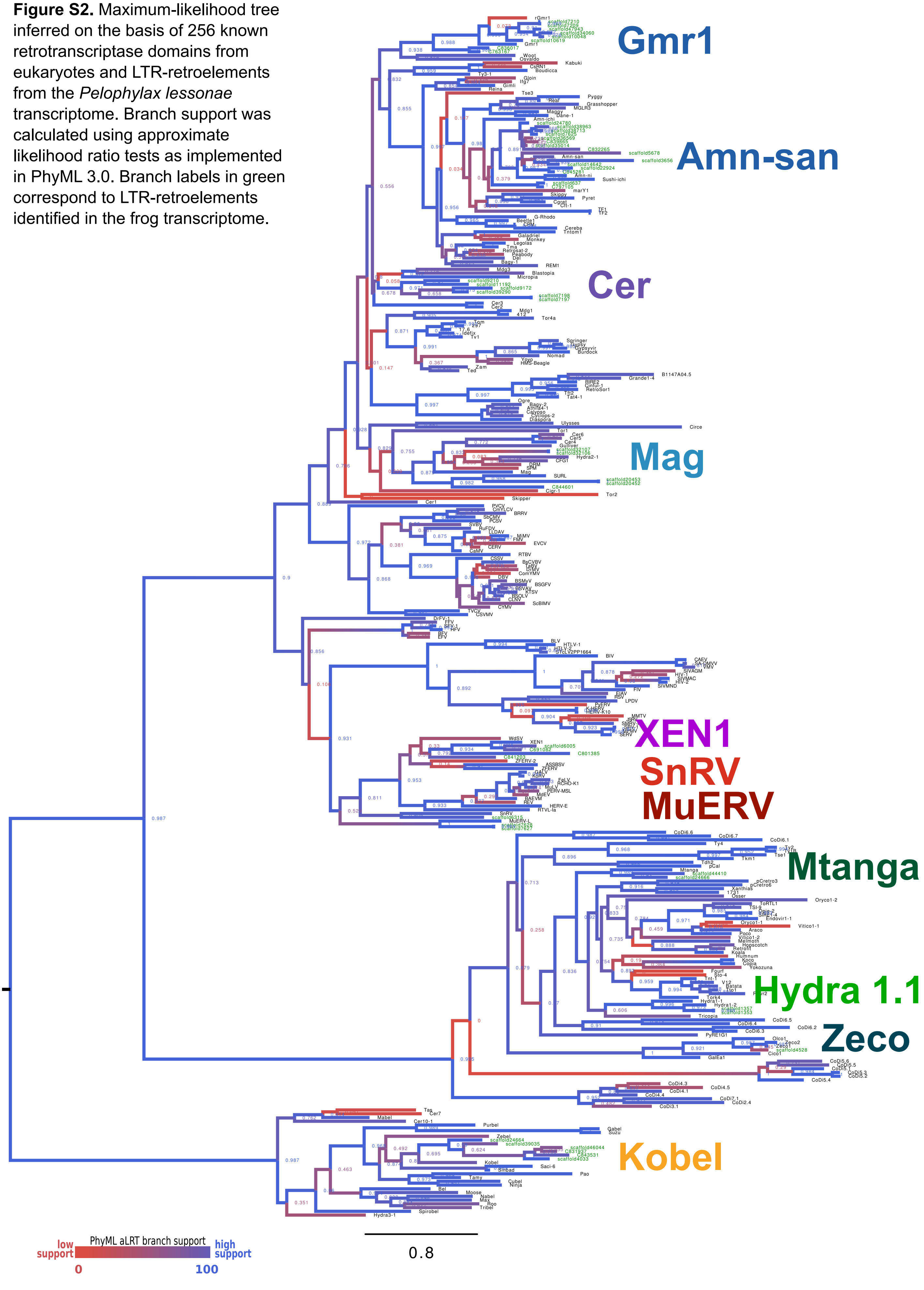

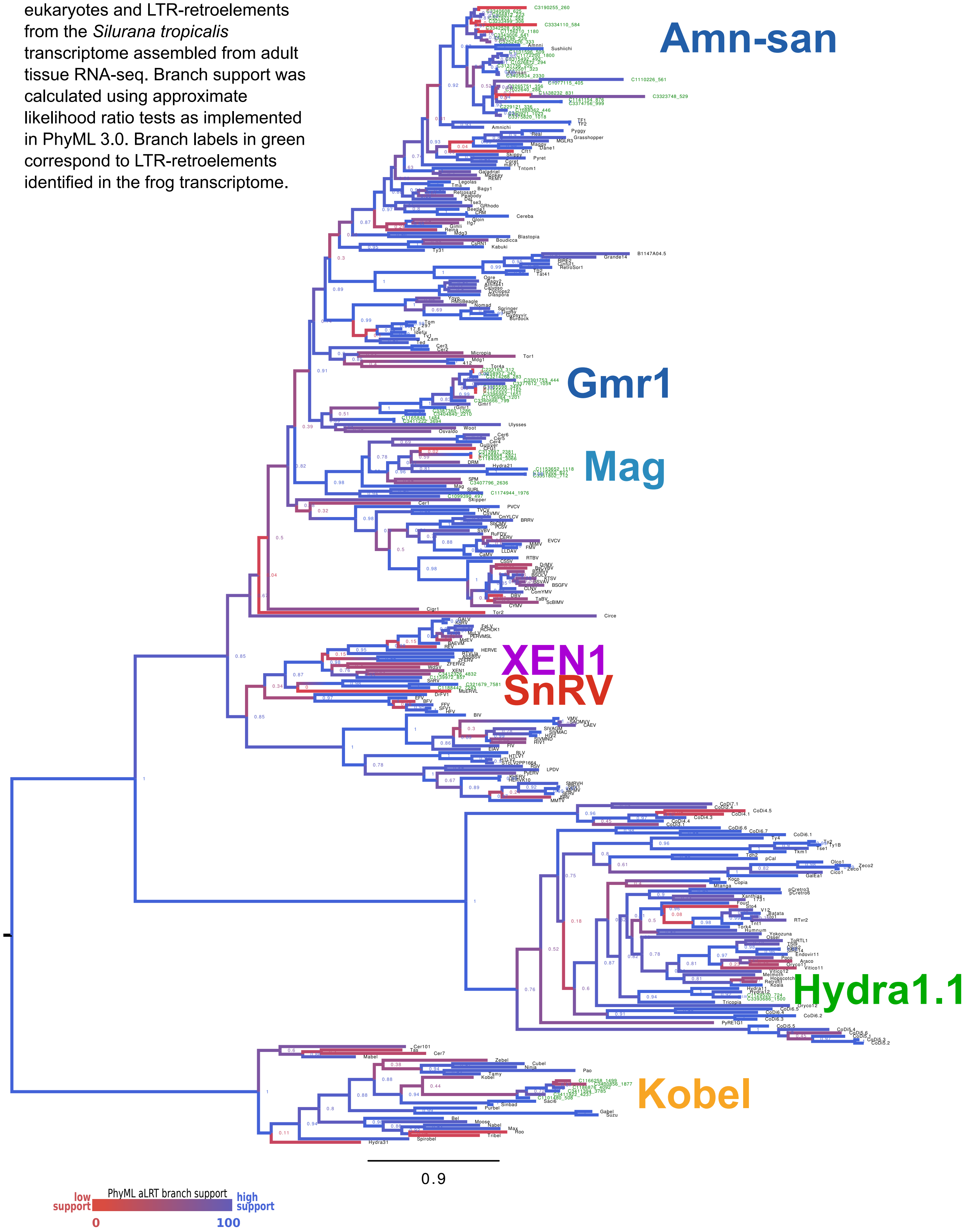


**Figure S3.** Maximum-likelihood tree inferred on the basis of 256 known retrotranscriptase domains from eukaryotes and LTR-retroelements from the *Silurana tropicalis* transcriptome assembled from adult tissue RNA-seq. Branch support was calculated using approximate likelihood ratio tests as implemented in PhyML 3.0. Branch labels in green correspond to LTR-retroelements identified in the frog transcriptome.

**Figure S4.** Maximum-likelihood tree inferred on the basis of 256 known retrotranscriptase domains from eukaryotes and LTR-retroelements from the *Cyclorana alboguttata* transcriptome. Branch support was calculated using approximate likelihood ratio tests as implemented in PhyML 3.0. Branch labels in green correspond to LTR-retroelements identified in the frog transcriptome.

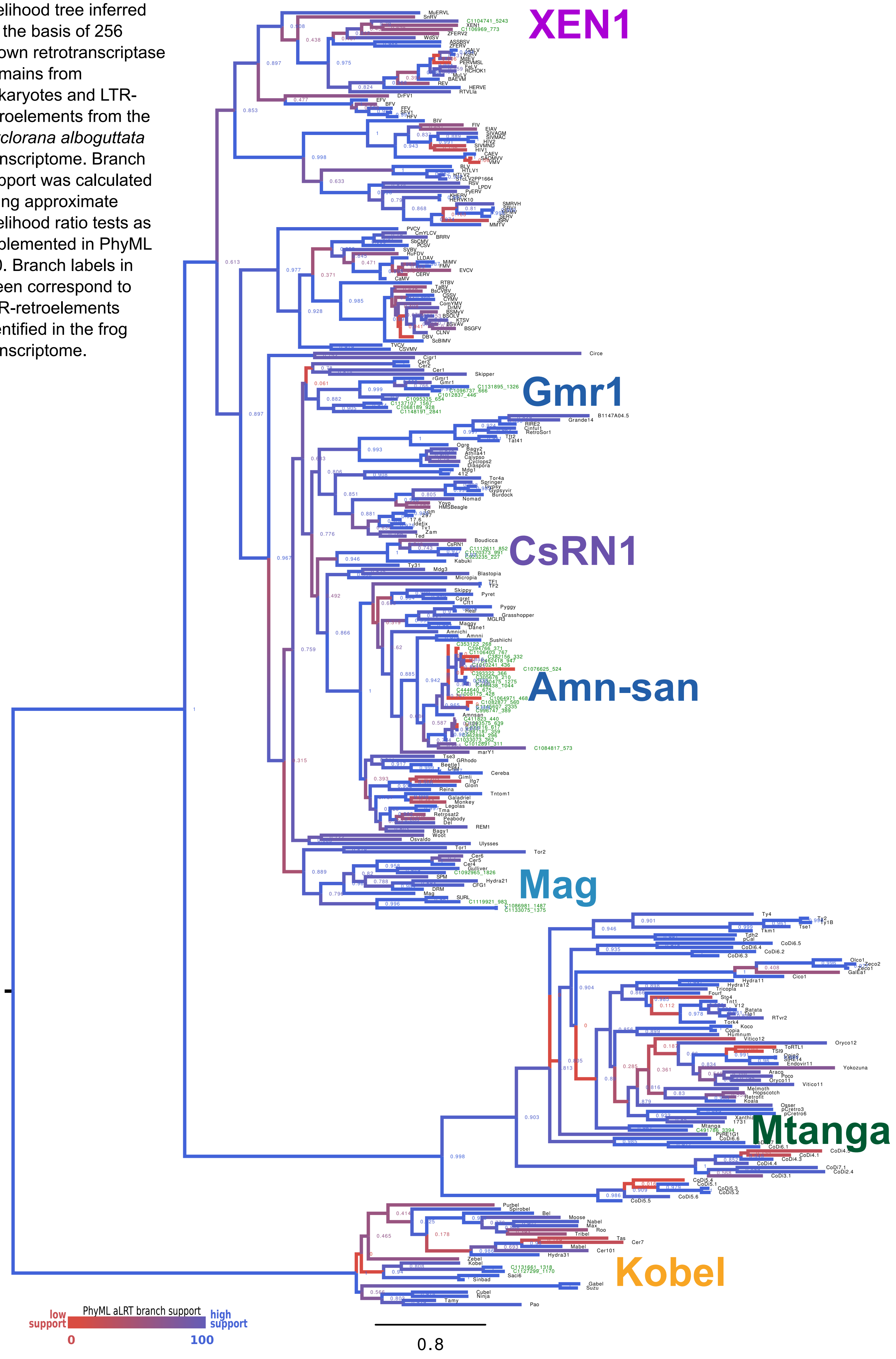

**Figure S5.** Maximum-likelihood tree inferred on the basis of 256 known retrotranscriptase domains from eukaryotes and LTR-retroelements from the embryonic *Silurana tropicalis* transcriptome. Branch support was calculated using approximate likelihood ratio tests as implemented in PhyML 3.0. Branch labels in green correspond to LTR-retroelements identified in the frog transcriptome.

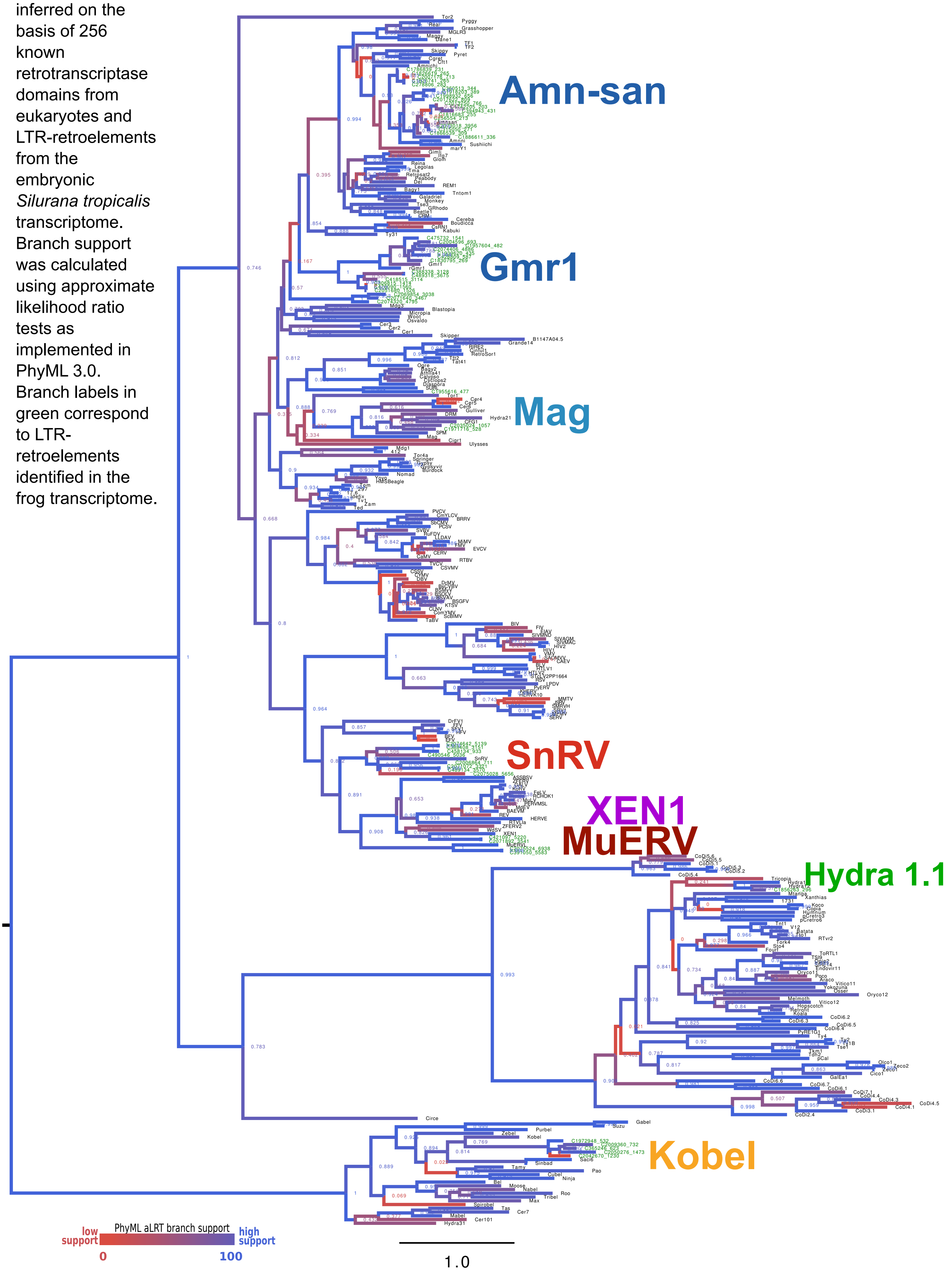

## 2. Element- and tissue-specific differential expression of LTR retroelements in frogs

### Element-specific expression

Normalized read counts (NRC) of eleven tissue-specific *P. lessonae* transcriptomes and five tissue-specific *S. tropicalis* transcriptomes are given in Table S1 and S2. Before testing for element-specific differences in expression, NRC values were transformed using a Box-Cox (POWER) transformation to obtain a normal distribution of the data:

$$Y = 1 + \frac{(X+\lambda_2)^{\lambda_1}-1}{\lambda_1 g^{\lambda_1 - 1}} \qquad \text{if} \qquad \lambda_1 \neq 0 \qquad (1)$$

$$Y = 1 + g\ln(X+\lambda_2) \qquad \text{if} \qquad \lambda_1 = 0 \qquad (2)$$

where $g$ is the geometric mean of the observations:

$$g = \left(\prod_1^n (X_i + \lambda_2)\right)^{\frac{1}{n}} \qquad (3)$$

For the eleven *P. lessonae* transcriptomes, NRC values (N=545) were transformed according to the formula: 1+((NRC-1.0)^0.081-1)/(0.081*204.188^-0.919). A $g$ value of 204.2 and a $\lambda_1$ value of 0.081 were determined after adding $\lambda_2 = -1$. The approximate 95% confidence interval for the power parameter $\lambda_1$ was 0.044 to 0.119, i.e. any power between these values could be a reasonable value for $\lambda_1$. A subsequent Shapiro-Wilks W test (SWT) confirmed the hypothesis that the transformed data follow a normal distribution (W=0.983; p=0.203). The same result was obtained for all tissue-specific sub-datasets (Table S3). Levene's test confirmed variance homogeneity for all data sets (p>0.05). The results of the ANOVA procedure and the Kruskal-Wallis test (KWT) revealed clear differences in the element-specific expression patterns for all tissues except heart (Table S1).

NRC values of five *Silurana* transcriptomes (N=307) were POWER transformed according to the formula: 1 + (NRC^-0.091-1)/(-0.091*64.1798^-1.091) with $g$=64.1798; $\lambda_1$=-0.092 (95% confidence limits: -0.149 and -0.036), and $\lambda_2$=0. The SWT statistics revealed no departure from normal distribution, either for the complete NRC data (W=0.985; p=0.593) or the tissue-specific datasets (Table S3). Levene's test revealed inhomogeneous variances for POWER transformed NRC values of kidney and liver. In contrast to the *Pelophylax* dataset, there are no significant differences in expression among the single elements in any of the tissues analyzed (Table S2).

**Table S1**. Numbers (first row) and arithmetic means of normalized read counts ± standard deviation (second row) of LTR retroelements detected in transcriptomes of different tissues obtained from *Pelophylax lessonae*. The test statistics presents the results of the ANOVA procedure (F/p), Kruskul-Wallis test (W/p), and Levene’s test for variance homogeneity (L/p). p values below 0.05 (red) indicate significant differences.

| **Family/Element** | **Brain** | **Eye** | **Heart** | **Intestine** | **Liver** | **Lung** | **Muscle** | **Skin** | **Stomach** | **Testis** | **Tongue** |
|---|---|---|---|---|---|---|---|---|---|---|---|
| **Bel/Pao** | | | | | | | | | | | |
| Kobel | 6<br>560.7±579.10 | 6<br>529.6±497.66 | 6<br>461.2±362.89 | 6<br>336.8±363.42 | 6<br>333.4±301.30 | 6<br>483.1±450.78 | 6<br>326.8±290.08 | 6<br>432.4±417.45 | 6<br>341.8±329.86 | 6<br>579.8±442.24 | 6<br>412.7±367.89 |
| **Ty1/Copia** | | | | | | | | | | | |
| Hydra1.1 | 2<br>3983.4±128.70 | 2<br>1549.8±35.34 | 2<br>889.9±16.6 | 2<br>1375.9±59.85 | 2<br>1073.4±40.38 | 2<br>1483.1±26.39 | 2<br>1031.8±24.30 | 2<br>2414.2±50.17 | 2<br>1113.0±30.37 | 2<br>988.0±17.58 | 2<br>2450.3±50.64 |
| Mtanga | 2<br>170.3±87.92 | 2<br>165.1±116.00 | 2<br>326.1±171.90 | 2<br>295.5±296.12 | 2<br>693.3±780.95 | 2<br>205.8±141.21 | 2<br>113.7±70.59 | 2<br>243.8±138.32 | 2<br>250.5±65.80 | 2<br>445.8±353.29 | 2<br>270.7±214.60 |
| Zeco | 1<br>327.1 | 1<br>418.3 | 1<br>140.8 | 1<br>167.8 | 1<br>152.0 | 1<br>240.0 | 1<br>418.9 | 1<br>249.3 | 1<br>286.3 | 1<br>313.8 | 1<br>208.8 |
| **Ty3/Gypsy** | | | | | | | | | | | |
| Amn-san | 13<br>584.3±1140.17 | 14<br>420.8±786.38 | 15<br>1107.5±2079.44 | 13<br>227.6±478.16 | 14<br>533.7±1512.29 | 13<br>424.0±900.81 | 13<br>312.8±503.66 | 13<br>447.6±1104.52 | 12<br>394.0±614.08 | 13<br>491.9±815.87 | 14<br>218.9±403.38 |
| Cer | 6<br>1569.8±2148.18 | 6<br>2416.9±3396.24 | 6<br>1990.3±2077.46 | 6<br>1053.9±1310.00 | 6<br>1248.9±1408.35 | 6<br>1631.2±1995.14 | 6<br>5165.6±5909.25 | 6<br>2536.2±3971.47 | 6<br>1102.3±822.49 | 6<br>4035.4±4634.31 | 6<br>2470.4±2909.41 |
| Gmr1 | 8<br>147.0±228.68 | 8<br>106.5±144.88 | 8<br>231.3±450.48 | 8<br>113.0±185.17 | 8<br>75.7±78.40 | 8<br>180.7±425.30 | 8<br>300.7±647.58 | 8<br>95.8±156.32 | 8<br>102.4±168.14 | 8<br>86.5±168.98 | 7<br>156.8±243.63 |
| Mag | 5<br>2887.4±3018.46 | 5<br>2512.4±2624.32 | 5<br>3081.4±3424.76 | 5<br>1404.8±1583.84 | 5<br>1126.8±1085.73 | 5<br>1415.8±1332.49 | 5<br>3821.2±4872.85 | 5<br>2372.2±2136.17 | 5<br>1114.4±1149.00 | 5<br>1668.1±1595.15 | 5<br>3319.4±3737.04 |
| **Retroviridae** | | | | | | | | | | | |
| MuERV | 2<br>2033.6±332.57 | 2<br>3350.0±466.30 | 2<br>368.8±58.34 | 2<br>3306.4±544.99 | 2<br>1977.5±335.54 | 2<br>1387.8±182.57 | 2<br>444.3±77.53 | 2<br>3046.6±372.7 | 2<br>2403.2±366.94 | 2<br>1624.4±212.65 | 2<br>1770.5±280.69 |
| SnRV | 1<br>72.1 | 1<br>29.3 | 1<br>18.1 | 1<br>23.8 | 1<br>15.1 | 1<br>16.1 | 1<br>83.5 | 1<br>26.4 | - | 1<br>26.6 | 1<br>5689.7 |
| XEN1 | 3<br>65.8±70.60 | 3<br>278.1±395.85 | 3<br>140.8±129.04 | 4<br>126.2±176.47 | 4<br>185.0±324.85 | 4<br>257.7±260.81 | 2<br>44.2±48.60 | 3<br>89.9±88.30 | 4<br>81.4±68.43 | 4<br>283.3±337.51 | 3<br>119.8±128.48 |
| **Total** | **49**<br>**999.2±1655.40** | **50**<br>**967.9±1731.58** | **51**<br>**1029.0±1847.86** | **50**<br>**597.7±997.82** | **51**<br>**619.6±1076.80** | **50**<br>**683.2±1055.47** | **48**<br>**1298.0±3037.36** | **49**<br>**983.9±1862.43** | **48**<br>**581.8±770.00** | **50**<br>**1014.2±2023.85** | 49<br>1087.7±1978.94 |
| **Test Statistics** | | | | | | | | | | | |
| ANOVA | **4.41/<0.001** | **3.92/0.001** | 1.94/0.067 | **3.90/0.001** | **3.39/0.003** | **3.31/0.003** | **2.82/0.011** | **3.63/0.002** | **4.10/0.001** | **4.37/<0.001** | **4.57/<0.001** |
| Kruskal-Wallis | **25.37/0.005** | **25.49/0.004** | 18.19/0.052 | **23.54/0.009** | **23.77/0.008** | **23.45/0.009** | **20.19/0.028** | **25.95/0.004** | **22.79/0.007** | **25.25/0.005** | **25.55/0.004** |
| Levene | 0.68/0.734 | 0.87/0.569 | 0.82/0.614 | 0.92/0.529 | 0.57/0.828 | 0.89/0.551 | 0.56/0.838 | 0.65/0.762 | 1.13/0.369 | 0.84/0.596 | 0.73/0.690 |

**Table S2**. Numbers (first row) and means of normalized read counts ± standard deviation (second row) of LTR retroelements detected in different tissues of *Silurana tropicalis* (NCBI SRA archive; Accession No. SRA051954). The test statistics presents the results of the ANOVA procedure (F/p), Kruskul-Wallis test (W/p), and Levene's test for variance homogeneity (L/p). p values below 0.05 (red) indicate significant differences. n.a. - not applicable.

| | brain | heart | kidney | liver | muscle |
|---|---|---|---|---|---|
| **Bel/Pao** | | | | | |
| Kobel | 5<br>60.1±64.60 | 6<br>100.7±138.98 | 5<br>143.9±170.21 | 4<br>193.3±335.18 | 6<br>149.2±175.64 |
| **Ty1/Copia** | | | | | |
| Hydra1.1 | 1<br>1316.8 | 2<br>422.3±586.67 | 2<br>425.1±598.17 | 2<br>1350.5±1908.06 | 2<br>787.7±1088.66 |
| **Ty3/Gypsy** | | | | | |
| Amn-san | 30<br>233.6±434.95 | 29<br>177.0±253.44 | 30<br>215.8±366.17 | 27<br>400.5±824.63 | 29<br>598.3±1492.82 |
| Gmr1 | 13<br>73.1±105.99 | 13<br>69.5±114.36 | 13<br>74.8±109.54 | 11<br>184.8±408.37 | 14<br>6025.8±22291.60 |
| Mag | 9<br>329.8±511.73 | 9<br>224.2±200.92 | 9<br>178.1±187.41 | 9<br>446.6±838.54 | 9<br>340.1±399.18 |
| **Retroviridae** | | | | | |
| SnRV | 2<br>26.4±8.11 | 2<br>20.2±3.17 | 2<br>17.8±13.88 | 1<br>66.7 | 2<br>71.6±42.20 |
| XEN1 | 2<br>146.8±197.90 | 2<br>70.3±84.56 | 2<br>46.9±64.17 | 1<br>35.2 | 2<br>183.5±217.31 |
| **Total** | **62<br>207.91±396.17** | **63<br>153.7±223.24** | **63<br>170.6±289.12** | **55<br>371.6±765.67** | **64<br>1683.6±10438.60** |
| **Test Statistics** | | | | | |
| ANOVA | 1.13/0.358 | 1.63/0.156 | n.a. | n.a. | 0.60/0.729 |
| Kruskal-Wallis | 5.91/0.433 | 7.40/0.286 | 5.19/0.519 | 3.67/0.721 | 4.37/0.627 |
| Levene | 1.53/0.184 | 1.82/0.112 | **3.47/0.006** | **3.34/0.008** | 1.15/0.348 |

## Tissue-specific expression

To answer the question whether LTR-REs are tissue specific expressed, relative NRC values ($NRC_{rel}$) were calculated for each transcriptome, dividing tissue-specific NRC values by tissue specific means ($NRC_{mean}$). Because $NRC_{rel}$ values were not normally distributed, all values were LOG transformed and subsequently tested for normality applying the SWT statistics (Table S3). If this transformation failed to result in a normal distribution, $NRC_{rel}$ values were POWER transformed as described above. In the *Pelophylax* dataset both LOG and POWER transformation failed to result in a normal distribution for Kobel, Cer, and Mag. Based on the transformed values it was tested for each element whether or not there were significant differences between the tissue-specific means and variances. Additionally, a Kruskal-Wallis test, which compares medians instead of means, was applied. This test is an alternative to a one-way ANOVA if the assumptions of variance homogeneity and normality are violated. Moreover, it is less sensitive to the presence of outliers than the ANOVA procedure. Significant tissue-specific differences in expression were obtained for Hydra1.1

and MuERV in the *Pelophylax* dataset and for Amn-san, Gmr1, and Mag in the *Silurana* dataset (Table S4, Figs. S6 and S7). In general, almost all elements have their lowest expression in muscle tissue of both *Pelophylax* and *Silurana* (Fig. S7). A further feature shared by both species is that Hydra1.1 and Amn-san are most heavily expressed in brain and heart, respectively.

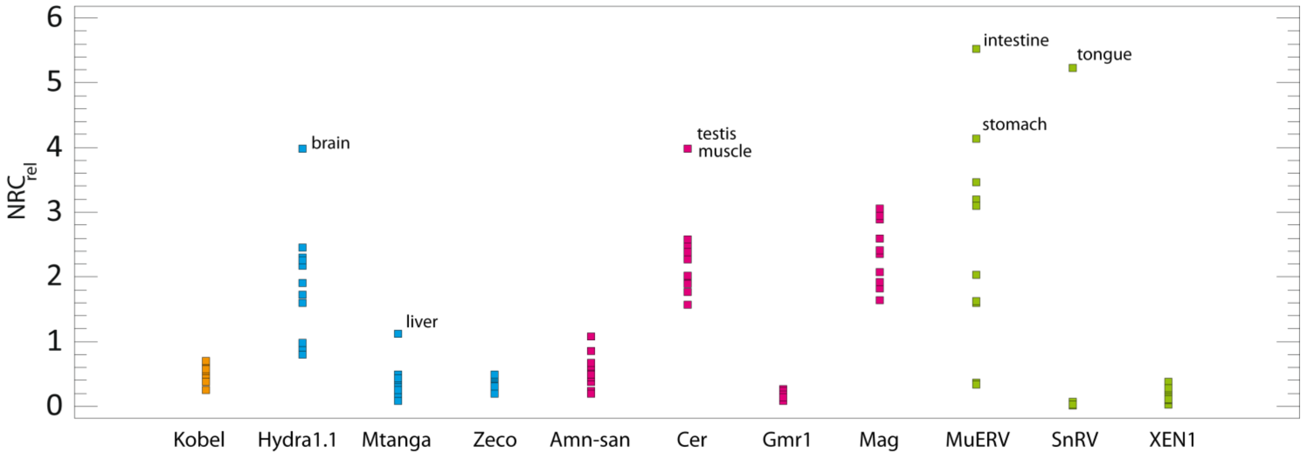


**Figure S6**. Tissue-specific expression (mean $NRC_{rel}$ values) of LTR retroelements in *P. lessonae*. LTR retroelement families are labeled by different colors (yellow: Bel/Pao, blue: Ty1/Copia, red: Ty3/Gypsy, green: Retroviridae).

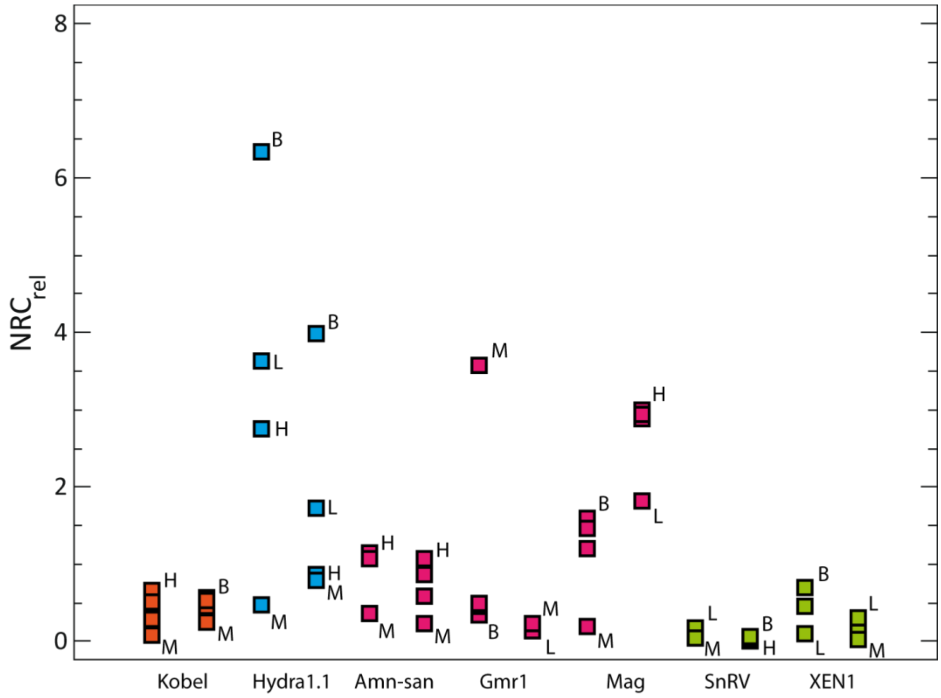


**Figure S7**. Differential expression (arithmetic means of relative NRC values) of LTR retroelements in four tissues (B: brain, H: heart, L: liver, M: muscle) of *S. tropicalis* (left points) and *P. lessonae* (right points). LTR retroelement families are labeled by different colors (yellow: Bel/Pao, blue: Ty1/Copia, red: Ty3/Gypsy, green: Retroviridae).

**Table S3.** Tissue specific means of NRC values, arithmetic mean± standard deviation of relative NRC values ($NRC_{rel}$), and results of Shapiro-Wilks tests (SWT) for Power transformed NRC values. N: number of NRC values, W: test statistics of SWT, p: significance level (levels below 0.05 would indicate departures from normal distribution).

| **Tissue** | ***Pelophylax*** | | | ***Silurana*** | | |
|---|---|---|---|---|---|---|
| | **$NRC_{mean}$** | **$NRC_{rel}$** | **SWT** | **$NRC_{mean}$** | **$NRC_{rel}$** | **SWT** |
| brain | 999 | N=49<br>1.0±1.66 | W=0.977<br>p=0.637 | 208 | N=62<br>1.0±1.91 | W=0.956<br>p=0.060 |
| eye | 968 | N=50<br>1.0±1.79 | W=0.966<br>p=0.274 | - | - | - |
| heart | 1029 | N=51<br>1.0±1.80 | W=0.973<br>p=0.454 | 154 | N=63<br>1.0±1.45 | W=0.959<br>p=0.080 |
| intestine | 598 | N=50<br>1.0±1.67 | W=0.963<br>p=0.207 | - | - | |
| kidney | - | - | - | 171 | N=63<br>1.0±1.70 | W=0.972<br>p=0.343 |
| liver | 620 | N=51<br>1.0±1.74 | W=0.972<br>p=0.431 | 372 | N=55<br>1.0±2.06 | W=0.981<br>p=0.735 |
| lung | 683 | N=50<br>1.0±1.54 | W=0.972<br>p=0.447 | - | - | - |
| muscle | 1298 | N=48<br>1.0±2.34 | W=0.972<br>p=0.468 | 1684 | N=64<br>1.0±6.20 | W=0.979<br>p=0.619 |
| skin | 984 | N=49<br>1.0±1.89 | W=0.980<br>p=0.722 | - | - | - |
| stomach | 582 | N=48<br>1.0±1.32 | W=0.954<br>p=0.092 | - | - | - |
| testis | 1014 | N=50<br>1.0±2.00 | W=0.981<br>p=0.779 | - | - | - |
| tongue | 1088 | N=49<br>1.0±1.82 | W=0.968<br>p=0.322 | - | - | - |

**Table S4**. Tissue-specific expression of LTR retroelements in *P. lessonae* and *S. tropicalis*. Test statistics are based on LOG-transformed relative NRC values or POWER transformed values (gray shaded lines). Values in parentheses are calculated on RNAseq data of individual No. PL74-2012, i.e. without the transcriptomes of heart and muscle. P-values below 0.05 (red) indicate significant differences. n.a.- not applicable.

| | ***Pelophylax*** | | | | | ***Silurana*** | | | | |
|---|---|---|---|---|---|---|---|---|---|---|
| **Element** | **N** | **ANOVA**<br>F/p | **Kruskal-Wallis**<br>H/p | **Shapiro-Wilk**<br>W/p | **Levene**<br>L/p | **N** | **ANOVA**<br>F/p | **Kruskal-Wallis**<br>H/p | **Shapiro-Wilk**<br>W/p | **Levene**<br>L/p |
| **Bel/Pao** | | | | | | | | | | |
| Kobel | 66<br>(54) | n.a.<br>(n.a.) | 2.78/0.986<br>(1.29/0.996) | **0.92/<0.001**<br>**(0.91/<0.001)** | 0.25/0.989<br>(0.29/0.965) | 26 | 2.08/0.119 | 6.86/0.144 | 0.959/0.390 | 1.24/0.323 |
| **Ty1/Copeia** | | | | | | | | | | |
| Hydra1.1 | 22<br>(18) | n.a.<br>(n.a.) | **20.80/0.022**<br>**(16.74/0.033)** | 0.91/0.052<br>(0.91/0.130) | **>10/<0.001**<br>**(5.51/<0.001)** | 9 | * | 1.93/0.748 | 0.834/0.05 | **>10/<0.001** |
| Mtanga | 22<br>(18) | n.a.<br>(n.a.) | 8.63/0.567<br>4.91/0.767 | 0.98/0.941<br>(0.96/0.583) | **>10/<0.001**<br>**(>10/<0.001)** | - | - | - | - | - |
| Zeco | 11<br>(9) | n.a.<br>(n.a.) | n.a.<br>(n.a.) | 0.98/0.951<br>(0.99/0.987) | n.a.<br>(n.a.) | - | - | - | - | - |
| **Ty3/Gypsy** | | | | | | | | | | |
| Amn-san | 147<br>(119) | 0.85/0.578<br>(0.72/0.677) | 9.68/0.469<br>(6.36/0.607) | 0.97/0.106<br>(0.97/0.211) | 0.19/0.997<br>(0.14/0.997) | 145 | **8.43/<0.001** | **23.67/<0.001** | 0.97/0.080 | 0.76/0.553 |
| Cer | 66<br>(54) | n.a.<br>(n.a.) | 7.94/0.634<br>(6.09/0.637) | **0.90/<0.001**<br>**(0.91/<0.001)** | 0.10/1.000<br>(0.07/1.000) | - | - | - | - | - |
| Gmr1 | 87<br>(71) | 0.34/0.968<br>(0.28/0.969) | 3.93/0.951<br>(2.65/0.954) | 0.97/0.162<br>(0.97/0.197) | 0.59/0.820<br>(0.64/0.738) | 64 | **2.78/0.035** | **11.64/0.020** | 0.983/0.788 | 1.29/0.247 |
| Mag | 55<br>(45) | n.a.<br>(n.a.) | 1.91/0.997<br>(1.64/0.990) | **0.90/<0.001**<br>**(0.92/0.003)** | 0.19/0.996<br>(0.12/0.998) | 45 | **2.71/0.044** | **8.77/0.067** | 0.972/0.488 | 0.59/0.673 |
| **Retroviridae** | | | | | | | | | | |
| MuERV | 22<br>(18) | n.a.<br>(n.a.) | **20.02/0.029**<br>**(15.68/0.047)** | 0.96/0.417<br>(0.94/0.317) | **>10/<0.001**<br>**(>10/<0.001)** | - | - | - | - | - |
| SnRV | 10 | n.a. | n.a. | 0.94/0.574 | n.a. | 9 | n.a. | 4.67/0.323 | 0.877/0.143 | **1.70/<0.001** |
| XEN1 | 37<br>(32) | 0.37/0.950<br>(0.29/0.962) | 4.01/0.947<br>(2.60/0.957) | 0.95/0.126<br>(0.95/0.166) | 0.26/0.984<br>(0.29/0.963) | 9 | n.a. | 1.07/0.900 | 0.964/0.831 | **>10/<0.001** |

### 3. Results of count data analyses

**Figure S8.** Analysis of RNA-Seq data obtained from tissue samples of *Pelophylax lessonae*. Heatmaps on the left side showing the expression data of LTR retrotransposon sequences in transcriptome as raw counts (a), counts normalized by library size (b), and counts given by DESeq's variance stabilizing transformation (c). Relationships between per-gene standard deviation (SD) and the rank of the mean of *P. lessonae* transcriptomic sequences are shown on the right side; (d) SDs for the shifted logarithm y=log2(n+1), and (e) for variance stabilizing transformation as implemented in DESeq (Anders and Huber 2010).

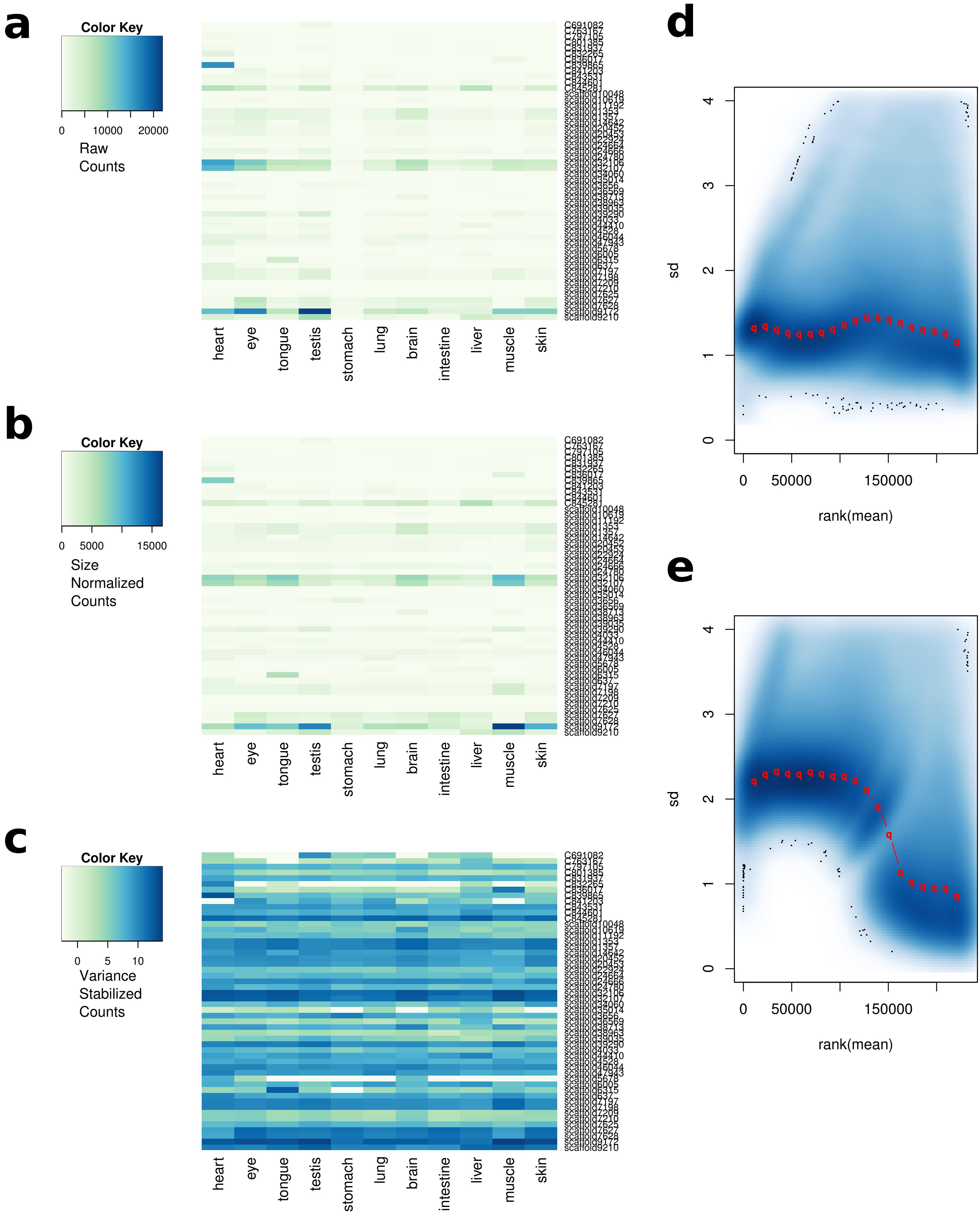

**Figure S9.** Analysis of RNA-Seq data obtained from tissue samples of *Silurana tropicalis*. Heatmaps on the left side showing the expression data of LTR retrotransposon sequences in transcriptome as raw counts (a), counts normalized by library size (b), and counts given by DESeq's variance stabilizing transformation (c). Relationships between per-gene standard deviation (SD) and the rank of the mean of *S. tropicalis* transcriptomic sequences are shown on the right side; (d) SDs for the shifted logarithm y=log2(n+1), and (e) for variance stabilizing transformation as implemented in DESeq (Anders and Huber 2010).

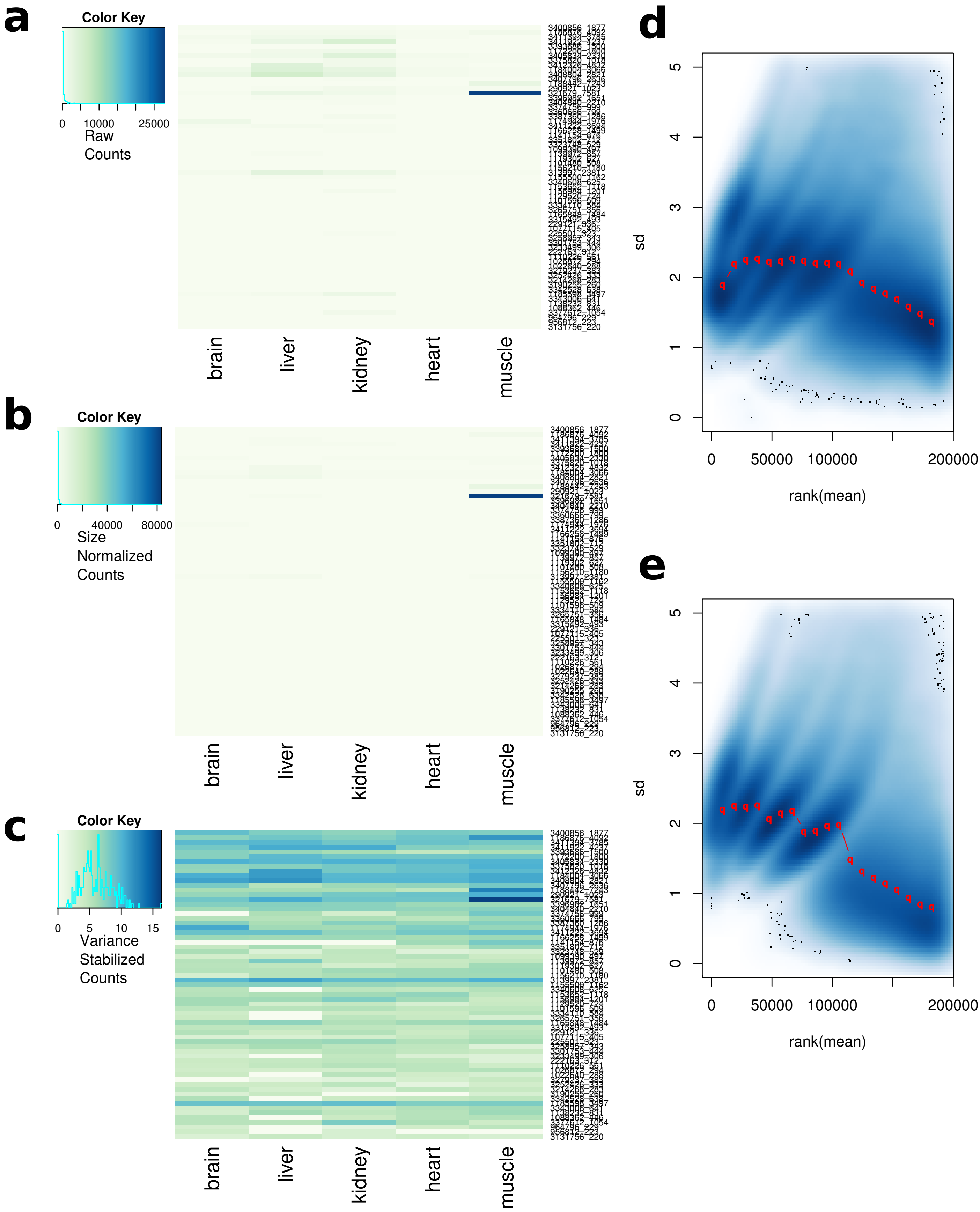

**Figure S10.** Analysis of RNA-Seq data obtained from skeletal muscle of eight *Cyclorana alboguttata* individuals. Heatmaps on the left side showing the expression data of LTR retrotransposon sequences in transcriptome as raw counts (a), counts normalized by library size (b), and counts given by DESeq's variance stabilizing transformation (c). Relationships between per-gene standard deviation (SD) and the rank of the mean of *C. alboguttata* transcriptomic sequences are shown on the right side; (d) SDs for the shifted logarithm y=log2(n+1), and (e) for variance stabilizing transformation as implemented in DESeq (Anders and Huber 2010).

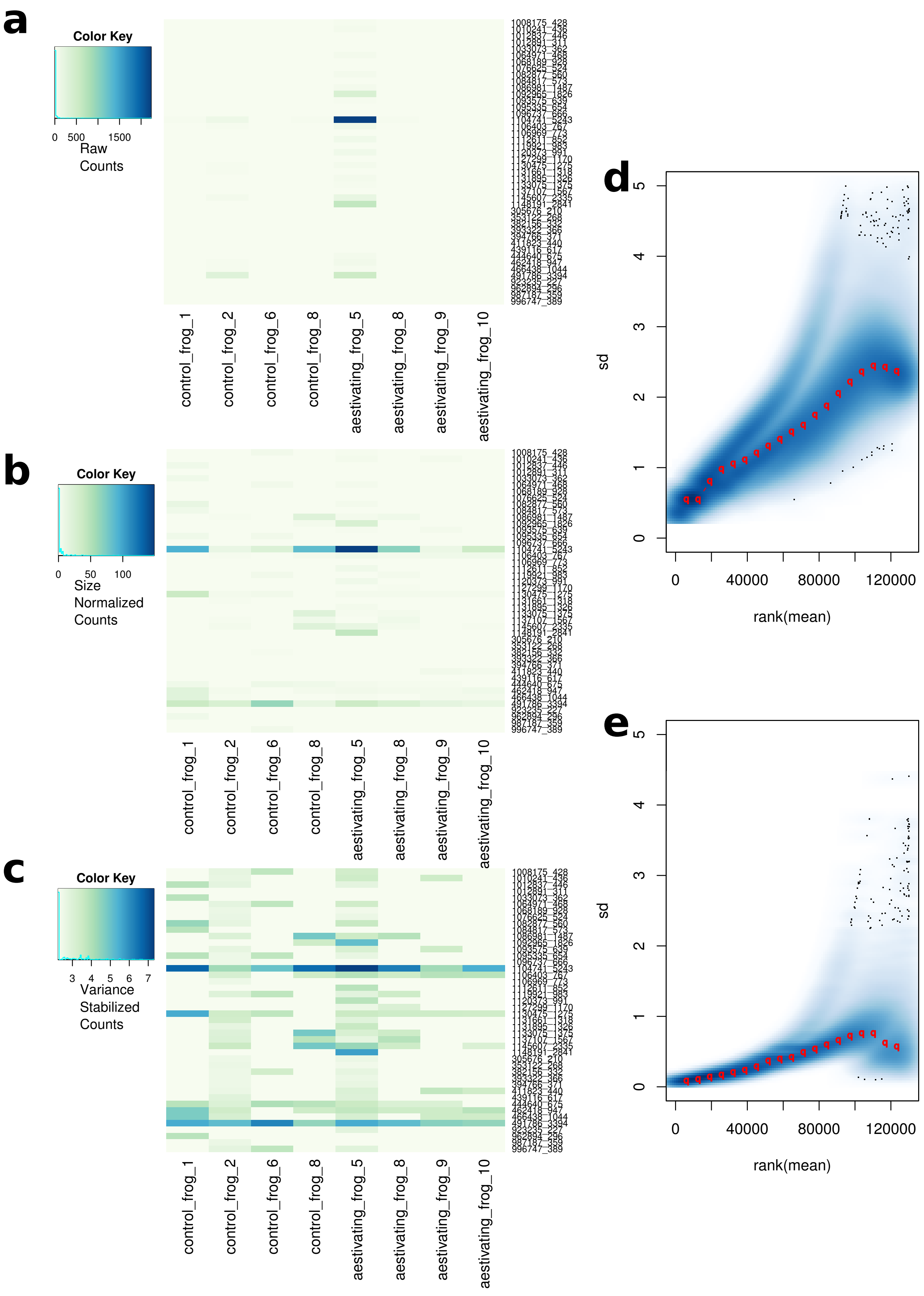

**Figure S11.** Analysis of RNA-Seq data obtained from different developmental stages (fertilized eggs, larvae) of *Silurana tropicalis*. Heatmaps on the left side showing the expression data of LTR retrotransposon sequences in transcriptome as raw counts (a), counts normalized by library size (b), and counts given by DESeq's variance stabilizing transformation (c). Relationships between per-gene standard deviation (SD) and the rank of the mean of *S. tropicalis* transcriptomic sequences are shown on the right side; (d) SDs for the shifted logarithm y=log2(n+1), and (e) for variance stabilizing transformation as implemented in DESeq (Anders and Huber 2010).

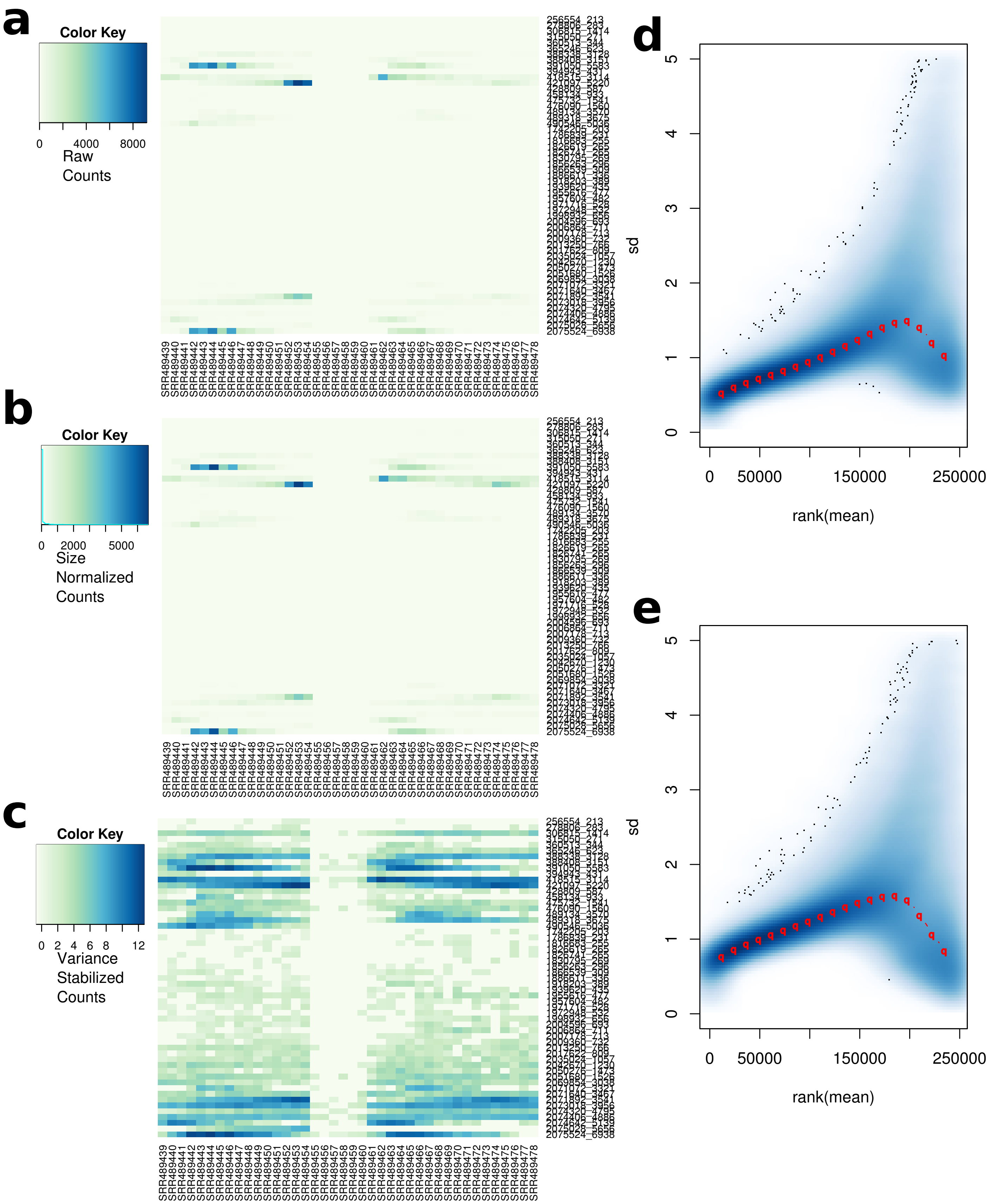

Supplement: Supplementary file 1 — Additional file 1: Supplemental material consists of supplementary methods, figures, and tables. (PDF 3 MB) [file 12864_2014_6338_MOESM1_ESM.pdf]
